# Intelligent documentation in medical education: can AI replace manual case logging?

**DOI:** 10.1093/jamiaopen/ooag067

**Published:** 2026-05-28

**Authors:** Nafiz Imtiaz Khan, Kiley Cleland, Vladimir Filkov, Roger Eric Goldman

**Affiliations:** Department of Computer Science, University of California, Davis, CA, United States; Department of Radiology, University of California, Davis, CA, United States; Department of Computer Science, University of California, Davis, CA, United States; Department of Radiology, University of California, Davis, CA, United States

**Keywords:** medical education, large language models, radiology, case logs, artificial intelligence

## Abstract

**Objective:**

This study investigates the feasibility of using large language models (LLMs) to automate procedural case log documentation in radiology training. We evaluate whether AI can replace manual logging, identify which procedure types are most challenging for extraction, and assess integration into clinical workflows.

**Materials and Methods:**

We retrospectively analyzed 414 curated radiology reports authored by nine interventional radiology residents between 2018 and 2024. A set of candidate models, including local (Qwen-2.5) and commercial (Claude-3.5), were tested under instruction and chain-of-thought prompting. Performance was measured by sensitivity, specificity, and F1-score, along with inference time and token efficiency to estimate operational cost.

**Results:**

Both local and commercial LLMs outperformed the standard benchmark. Qwen-2.5 achieved F1-scores of 86.66 with chain-of-thought prompting, while Claude-3.5-Haiku reached an F1-score of 86.89%. Commercial inference delivered sub-2s latency and concise outputs, while local deployment traded speed for lower recurring cost. Automation could save over 35 hours of manual annotation per resident annually.

**Discussion:**

LLMs can provide a scalable and accurate solution for radiology case log documentation. Optimizing for procedure-specific challenges and ensuring seamless integration with existing systems will be essential. Future work should validate across larger, multi-institution datasets and explore additional prompting strategies.

**Conclusion:**

LLMs show promise for automating radiology case log documentation, potentially reducing resident clerical burden. However, this single-institution feasibility study underscores the need for broader validation across diverse institutions, assessment of real-world workflow integration, and safeguards against misclassification before clinical adoption.

## Introduction

Accurate and comprehensive clinical activity documentation is paramount for medical education and professional practice.^1^ Among the most critical artifacts in this process are physician case logs, which serve as structured records that detail the types and frequencies of procedures performed by individual clinicians. These logs are integral to multiple high-stakes functions. They provide evidence for board certification, serve as official documentation for credentialing and privileging within hospitals, and support quality assurance and performance evaluations.^2–5^ During medical training, case logs are especially vital, enabling program directors to monitor resident progress, ensure exposure to a sufficient variety and volume of procedures, and identify areas needing remediation. Importantly, these logs also underpin institutional compliance with accreditation requirements, such as those mandated by the Accreditation Council for Graduate Medical Education (ACGME) (https://www.acgme.org/).

However, the current reality of case log documentation in many specialties, particularly radiology, is that the process remains highly manual, fragmented, and inefficient. Radiology trainees are typically required to self-report the procedures they perform by entering data into spreadsheets or institutional logging systems.^6^ Each entry often includes detailed metadata, such as the type of imaging modality, the anatomical site, the nature of the procedure, and whether it was performed independently or under supervision. According to Cox et al.,^7^ general residents spend a median of 23.7 hours per week—nearly 38% of their duty hours—engaged with the electronic health record (EHR). While this reflects overall documentation burden rather than case log entry specifically, it highlights how clerical tasks consume significant trainee time. Given that a radiology resident may perform well over 1600 (typically 1500 to 1800) procedures throughout their residency, the cumulative documentation burden is substantial. This manual process consumes valuable time that could otherwise be devoted to clinical learning or scholarly activity. In addition, manual effort introduces considerable potential for errors and inconsistencies in the documentation. Moreover, the absence of real-time feedback mechanisms limits the utility of these logs as tools for formative assessment.

In an era where natural language processing (NLP) and machine learning (ML) technologies, particularly large language models (LLMs), have made significant advancements,^8–10^ there exists a compelling opportunity to reimagine how procedural case logs are created.^11–19^ Radiology reports, routinely authored by radiology residents and attendings, provide comprehensive narrative summaries of diagnostic imaging encounters and are inherently rich in procedural detail. These reports are routinely produced as part of standard clinical documentation and are stored in digital or semi-structured formats within Electronic Health Records (EHRs),^20^ Radiology Information Systems (RIS),^21^ or Picture Archiving and Communication Systems (PACS).^22^ Using LLMs to automatically extract structured procedural data from these unstructured narratives could generate accurate procedural case logs with minimal human intervention. Such automation promises not only to alleviate administrative burden but also to enhance the accuracy, completeness, and timeliness of documentation. At the same time, prior experience with clinical AI tools demonstrates the risks of overclaiming performance without rigorous validation,^23^^,^^24^ underscoring the importance of cautious evaluation in this domain.

While the theoretical potential of LLMs in this space is enticing, several important objectives motivate this study. First, we assess whether AI can effectively automate procedural case log documentation in radiology training, examining the feasibility and performance of using LLMs to convert narrative radiology reports into structured, loggable entries. Second, we investigate whether performance varies across procedure categories, identifying which categories are difficult to classify, and explored the linguistic or structural reasons behind these disparities. Finally, we examine the translational challenges of real-world deployment, including cost, and processing time.

In sum, this is the first work that explores the intersection of artificial intelligence and procedural documentation in medical education by systematically evaluating the feasibility of automation, the variation in accuracy across procedure categories, and the practical considerations for integration into clinical and educational workflows. Ultimately, we envision a future in which intelligent systems reduce administrative burden, improve documentation quality, and allow medical trainees to devote more attention to patient care and professional development.

## Methodology

This retrospective study was approved by the institutional review board. The requirement of informed consent for inclusion in the study was waived. No protected health information is included in the reported results.

### Data collection

We retrospectively collected a dataset of raw radiology reports from a tertiary-care academic medical center, spanning the period from October 2018 through September 2024. These reports were authored by one of nine current or former Interventional Radiology (IR) residents. From this larger pool (36,659 reports across 301 unique exam codes, including 162 distinct procedural codes), we curated a subset of 414 reports corresponding to 39 representative IR procedures. For each procedural exam code, at least one corresponding radiology report was selected for manual labeling. If a code was associated with two reports, both were labeled. For codes with three or more associated reports, a random sample of three reports was selected for labeling. This ensured inclusion across the full range of required categories, but we acknowledge the small sample size per category as a limitation.

### Data annotation

Following data collection, we conducted a structured annotation process to determine which unique radiology procedures were mentioned in each report. This task involved identifying the presence or absence of any of 39 predefined radiology procedures, which were based directly on the American Board of Radiology (https://www.theabr.org/) Case Log. The ABR case log was selected due to the diversity of procedure categories, which closely mirrors individual hospital case log requirements for privileges and is more granular than the ACGME logs. Procedures were organized into three high-level categories: Vascular Diagnosis (8 procedures), Vascular Intervention (15), and Non-Vascular Intervention (16). The complete list is shown in Table 4.

Two independent annotators reviewed each report to assign binary labels for each of the 39 procedures: one interventional radiology trainee and one board-certified interventional radiologist with six years of post-graduate experience serving as an Assistant Professor at UC Davis Health. A label of 1 was assigned if a procedure was explicitly or implicitly mentioned in the report, and 0 otherwise. The default label was 0, and annotators marked 1 only when confident a procedure was present. To assess labeling consistency, we calculated inter-annotator agreement using Cohen’s Kappa, which accounts for chance agreement.^25^^,^^26^ The observed agreement was κ=0.896, which indicates strong agreement between the annotators. Discrepancies were resolved by consensus, and the adjudicated labels served as the reference standard.

This labeling scheme operates at the procedure level: for each report, a binary label was independently assigned for each of the 39 procedures, resulting in 16,146 binary classification instances across the full dataset. The task, therefore, is multi-label binary classification; not document-level categorization.

### Data pre-processing

We applied lightweight pre-processing to make the reports suitable for model inference. All text was lowercased to reduce case-sensitivity bias. Non-informative sections such as “attestation” and “addendum” were removed, as they typically contain administrative or author-identifying information irrelevant to procedural content. Also, non-ASCII characters were stripped to ensure tokenization compatibility.

### LLM selection

Our goal was to evaluate a set of local and commercial LLMs on their ability to extract procedural data from radiology reports. Importantly, this was an *inference-only* study: no model training or fine-tuning was performed. All models were applied directly in zero-shot mode to the dataset, so a train/validation/test split was not applicable.

#### Selection criteria

##### Privacy and compliance

Given the sensitive nature of clinical data, model deployment was constrained to HIPAA-compliant environments. For commercial evaluation, we used AWS Bedrock, which provides HIPAA-eligible infrastructure under a Business Associate Agreement (BAA), ensuring that clinical data does not leave the institution’s compliant cloud boundary. This requirement precluded the use of some prominent commercial models, including OpenAI’s GPT-4 (ChatGPT) and Google’s Gemini, as their respective API platforms were not covered under our institutional data use agreements at the time of this study.

###### Model type

We selected *instruction-tuned* models, optimized for following prompts and returning structured responses,^27^ since these are well-suited for extraction tasks.

###### Hardware constraints

Selection was also informed by available compute resources. Our local secure server had limited GPU memory, excluding extremely large models. Server specifications are listed in Table B.1 of Appendix B, available as supplementary data at [*JAMIA Open*] online.

###### Model version and size

When multiple versions existed, we chose the largest feasible variant within our hardware and compliance constraints. For example, LLaMA 3.3-70B was used locally, while the most performant model on AWS Bedrock at the time (Claude-3.5-Haiku, February 2025) was selected commercially.

#### Selected models

Six models were evaluated: five open-source/local and one commercial. These included Mixtral-8x7B, LLaMA 3.3-70B, Qwen 2.5-72B, MedLLaMA-2-7B, LLaMA-3-Med42-70B, and Claude-3.5-Haiku. Model details are provided in the Appendix E, available as supplementary data at [*JAMIA Open*] online. No additional fine-tuning was performed; all models were evaluated as released by their developers.

### Benchmark definition (crosswalk)

For comparison, we leveraged the “crosswalk” benchmark,^28^ which is a rule-based mapping between exam codes or CPT codes and predefined procedure categories. For example, CPT code 75625 corresponds to “Lower Extremity Angiography.” This approach, while widely used and straightforward, is limited by coding variability and inability to capture multi-procedure reports. As such, it offers high specificity but reduced sensitivity, and serves as a baseline against which LLM outputs can be compared.

### Prompt engineering

Two prompting strategies were tested with each model: (i) *Instruction Prompting*, where the task was explicitly stated and the model was expected to return the output directly,^27^^,^^29^ and (ii) *Chain-of-Thought (CoT)* prompting, where the model was guided to reason step-by-step before generating the final answer.^30^^,^^31^ CoT was implemented via a standardized template enforcing stepwise reasoning and a consistent output structure. No few-shot examples or fine-tuned prompts were introduced, to maintain consistency across models.

### Pipeline implementation

We developed a modular pipeline in Python to support reproducible evaluation. The pipeline managed preprocessing, model querying (local via Ollama,^32^ cloud via AWS Bedrock), and logging of results. Outputs were parsed into structured JSON and saved with metadata (model, prompt type, report ID, timestamp) for traceability. This ensured consistent handling across all models and prompt strategies.

### Model evaluation

We evaluated model outputs against the adjudicated ground truth labels. Metrics included True Positive (TP), True Negative (TN), False Positive (FP), False Negative (FN), Sensitivity, Specificity, and F1-score.^33^^,^^34^ We also tracked inference time and token counts to estimate computational and financial costs. Performance was summarized across all 39 procedure categories, with per-category error analysis conducted to identify systematically difficult categories. All evaluations were conducted under consistent experimental conditions, following best practices such as STARD-AI for transparent reporting in medical AI.^35^

Importantly, LLMs in this study were used exclusively as classifiers applied to clinician-authored radiology reports. No reports were generated by LLMs. Model outputs, structured binary indicators for each of the 39 procedures, were compared against human-adjudicated ground truth labels.

## Results

### Automating procedural case log documentation

We evaluated model performance using a test set of 414 radiology reports annotated by independent reviewers. The inter-annotator agreement, measured using Cohen’s Kappa, was 0.896—indicating strong consistency. Disagreements were resolved through consensus to establish the final ground truth used for evaluation.

Table 1 presents a detailed comparison between the Crosswalk benchmark, the top-performing local model (*Qwen-2.5:72B*), and the top-performing commercial model (*Claude-3.5-Haiku*) across two prompting strategies—Instruction Prompting (IP) and Chain-of-Thought (CoT)—as well as across three procedural modality categories. The performance of other local models is provided in Table C.2 in the Appendix C, available as supplementary data at [*JAMIA Open*] online.

**Table 1.** Performance of the crosswalk benchmark compared to the top-performing local and commercial language model.

Model Type	Model-Name	Prompting Method	Modality	TP	TN	FP	FN	Sensitivity (%)	Specificity (%)	F1-Score (%)
**Benchmark**	Cross-Walk	NA	All	451	15364	93	238	65.46	99.40	**73.15**
			VascularDiagnosis	143	3065	23	81	63.84	99.26	73.33
			VascularIntervention	157	5906	38	109	59.02	99.36	68.11
			NonVascularIntervention	151	6393	32	48	75.88	99.50	79.06
**Local**	Qwen-2.5:72B	IP	All	649	15174	283	40	94.19	98.17	**80.08**
			VascularDiagnosis	219	3068	20	5	97.77	99.35	94.60
			VascularIntervention	247	5803	141	19	92.86	97.63	75.54
			NonVascularIntervention	183	6303	122	16	91.96	98.10	72.62
		CoT	All	627	15326	131	62	91.00	99.15	**86.66**
			VascularDiagnosis	214	3071	17	10	95.54	99.45	94.07
			VascularIntervention	242	5868	76	24	90.98	98.72	82.88
			NonVascularIntervention	171	6387	38	28	85.93	99.41	83.82
**Commercial**	Claude-3.5-Haiku	IP	All	633	14961	496	56	91.87	96.79	**69.64**
			VascularDiagnosis	215	3067	21	9	95.98	99.32	93.48
			VascularIntervention	230	5737	207	36	86.47	96.52	65.43
			NonVascularIntervention	188	6157	268	11	94.47	95.83	57.41
		CoT	All	613	15348	109	76	88.97	99.29	**86.89**
			VascularDiagnosis	210	3069	19	14	93.75	99.38	92.71
			VascularIntervention	228	5905	39	38	85.71	99.34	85.55
			NonVascularIntervention	175	6374	51	24	87.94	99.21	82.35

Bold values indicate the highest F1-Score achieved within each model category (Benchmark, Local, and Commercial) across prompting strategies and modalities.

The Crosswalk benchmark, which relies solely on structured metadata from radiology reports, displays excellent specificity across all categories (overall specificity: 99.40%). This confirms its conservative design, prioritizing false positive minimization. However, its sensitivity remains low (65.46%), indicating substantial under-detection of documented procedures, particularly those mentioned only in free-text. This trade-off is most pronounced in the *Vascular Intervention* category, where sensitivity drops to 59.02%, suggesting that the benchmark fails to capture many interventional procedures lacking structured documentation. While *Vascular Diagnosis* and *Non-Vascular Intervention* fare slightly better (sensitivity: 63.84% and 75.88%, respectively), they still reflect the benchmark’s inherent limitation in sensitivity.

In contrast, the local model *Qwen-2.5:72B* demonstrates marked improvements in both sensitivity and overall performance when augmented with prompting strategies. Under Instruction Prompting, Qwen achieves a sensitivity of 94.19% and specificity of 98.17%, yielding a notable gain of +28.73% points in sensitivity over the Crosswalk baseline, without substantially compromising specificity. This results in an F1-score of 80.08—a lift over the benchmark. CoT prompting further improves the balance between sensitivity and specificity, with Qwen attaining a higher F1-score (86.66%) among all local configurations. This is particularly driven by CoT’s effectiveness in reducing false positives in the *Non-Vascular Intervention* category (FP: 38 vs 122 under IP), highlighting its ability to support more nuanced reasoning in complex textual contexts.

The commercial model *Claude-3.5-Haiku* similarly benefits from both prompting paradigms. With Instruction Prompting, it achieves a sensitivity of 91.87%, specificity of 96.79%, and F1-score of 69.64. However, CoT prompting enhances performance further: the F1-score rises to 86.89; the highest across all models and configurations—accompanied by an improved specificity of 99.29%. These results suggest that Claude’s CoT-based reasoning is particularly best-suited for the intricacies of radiology language. On a per-modality basis, Claude demonstrates outstanding sensitivity in *Vascular Diagnosis* (95.98% under IP) and achieves the most consistent performance across all categories under CoT.

A broader trend across all evaluated models is that *Vascular Diagnosis* consistently yields the highest sensitivity and F1-scores. This is likely due to the use of standardized terms and clear procedural language in the diagnostic procedural categories, which facilitates model understanding. On the other hand, *Vascular Intervention* remains the most challenging category, especially for the Crosswalk benchmark, but also for models operating under Instruction Prompting. The improved results from CoT prompting—particularly for Qwen (F1: 82.88) and Claude (F1: 85.55)—highlight the benefit of step-by-step reasoning in interpreting diverse and often implicit procedural descriptions typical of interventional reports.

Overall, these findings underscore the limitations of metadata-only benchmarks for procedure detection and illustrate the potential of LLMs, especially when equipped with effective prompting strategies, to more accurately and comprehensively extract procedural information from unstructured clinical text.

### Variation across procedure types

Table D.4 in appendix D, available as supplementary data at [*JAMIA Open*] online reports the number of false positives (FP) and false negatives (FN) committed by three algorithms—the Crosswalk benchmark, the top-performing commercial model with CoT prompting (*Claude-3.5-Haiku*), and the top-performing local model with CoT prompting (*Qwen-2.5:72B*)—across 39 radiological procedures. This fine-grained error analysis provides insight into procedure-level performance and helps assess whether certain procedures are consistently harder to detect

Compared to the Crosswalk benchmark (652 FNs, 244 FPs), both LLMs achieve significant reductions in false negatives: *Claude-3.5-Haiku* reduces FNs to 76, and *Qwen-2.5:72B* to 62. However, this gain in sensitivity comes at the cost of increased false positives: Claude reports 109 FPs, while Qwen reports 131—still lower than Crosswalk’s FP count.

#### Poorly classified procedures

Certain procedures remain challenging for all models, with high error rates suggesting inherent complexity or ambiguity. Notably, Procedure 23 (Other, Vascular) and Procedure 39 (Other, NonVascular) exhibit the highest cumulative errors across all three systems. For example, Qwen reports 45 false positives on Procedure 23 and both models struggle with Procedure 39, where errors remain concentrated due to overlapping semantics and subtle contextual clues.

#### Well-classified procedures

Several procedures such as Procedure 2, Procedure 11, Procedure 18, and Procedure 28 are consistently well-classified across all models. These likely benefit from clearer textual cues or isolated contexts. Procedures like Procedure 12, Procedure 16, and Procedure 30 show modest improvements in both FP and FN rates with the LLMs compared to Crosswalk.

#### Model-specific patterns

*Claude-3.5-Haiku* maintains a relatively balanced error profile with moderate FPs and low FNs. In contrast, *Qwen-2.5:72B* demonstrates aggressive prediction behavior—especially in Procedure 23—resulting in more FPs but fewer FNs overall. This behavior may be attributed to the model’s higher confidence threshold or broader generalization strategy.

### Practical deployment considerations

Table 2 presents descriptive statistics—including maximum, minimum, mean, and standard deviation—for inference time and generated token count for the Claude-3.5 Haiku and Qwen2.5:72B models across the two prompting strategies: IP and CoT. The reported descriptive statistics reflect per-procedure identification. Therefore, multiplying these values by 39 yields the corresponding per-report estimates.

**Table 2.** Per procedure identiciation cost of running the language model in relation to inference time and generated tokens.

Model Name	Model Type	Prompting Method	Inference Time (Second)				Generated Tokens (Count)			
			Max	Min	Mean	Std Dev	Max	Min	Mean	Std Dev
**Qwen2.5:72B**	Local	IP	20.00	4.00	9.70	2.15	127	27	63.01	14.39
		CoT	48.00	9.00	13.47	3.23	111	26	53.98	12.27
**Claude-3.5-Haiku**	Commercial	IP	5.00	1.00	1.97	0.70	67	13	40.26	9.77
		CoT	15.00	3.00	6.97	2.15	80	10	29.79	9.60

Additionally, the cost metrics (inference time and generated token) of the additional local models are presented in Table C.3 in Appendix C, available as supplementary data at [*JAMIA Open*] online.

#### Inference time

Inference Time/Latency is a key operational constraint in any real-world deployment. Among the evaluated configurations, the commercial model *Claude-3.5-Haiku* using Instruction Prompting (IP) demonstrates the fastest average per-procedure inference time—1.97 seconds—with a minimum of just 1 second and maximum of 5 seconds. This near-instant responsiveness positions Claude as a highly practical solution for interactive and high-volume use cases. In contrast, the local model *Qwen-2.5:72B*, while effective in terms of accuracy, demonstrates a higher computational burden. Under Chain-of-Thought (CoT) prompting, it reaches a mean inference time of 13.47 seconds per procedure, with outliers extending to 48 seconds. This latency may constrain scalability, particularly in batch-processing or real-time scenarios, unless sufficient parallelization or hardware resources are available.

#### Token efficiency and output volume

Token generation affects not only the cost of the model, especially in commercial settings, but also the interpretability and processing overhead. *Claude-3.5-Haiku* again leads in efficiency, with CoT prompting producing an average of 29.79 tokens per procedure, and IP generating slightly longer responses at 40.26 tokens. By contrast, Qwen’s IP configuration yields significantly longer outputs (mean: 63.01 tokens), suggesting more verbose justifications. Although longer responses can provide more rationale, they also increase downstream token-related costs and may slow user consumption of the output. Thus, from a human-in-the-loop perspective, Claude’s concise formatting is more aligned with practical review and integration.

Table 3 details the estimated cost of running the Qwen2.5:72B model in a local deployment environment, providing both per procedure and per report costs in USD for the two strategies—IP and CoT. These cost estimates are based on the model’s average inference time, which serves as a practical proxy for computational resource utilization in local settings. Specifically, the cost of running the local model is computed by multiplying the per-procedure inference time by the estimated per-second operational expense of the local server. This estimate incorporates hardware depreciation, electricity consumption, and general infrastructure overhead. The detailed cost breakdown for operating the local system is outlined below.

**Table 3.** Comparative cost of running the most performant models in commercial (Claude-3.5-Haiku via AWS Bedrock) and local (Qwen2.5:72B) environments.

Model	Prompting Method	Category	Input/Time	Output Tokens	Cost (USD)
**Claude-3.5-Haiku (Commercial, AWS Bedrock)**	IP	Procedure	837.41 tokens	40.05 tokens	$0.0008
		Report	32658.96 tokens	1561.79 tokens	$0.0324
	CoT	Procedure	1232.77 tokens	30.84 tokens	$0.0011
		Report	48077.96 tokens	1202.84 tokens	$0.0433
**Qwen2.5:72B (Local Server)**	IP	Procedure	9.70 s	—	$0.00094
		Report	378.30 s	—	$0.03670
	CoT	Procedure	13.47 s	—	$0.00131
		Report	525.33 s	—	$0.05096

**Table 4.** Categorization of procedures into predefined modality groups: vascular diagnosis, vascular intervention, and non-vascular intervention.

Count	Procedure Description	Modality
**1**	Computed Tomography Angiography (CTA)	Vascular Diagnosis
**2**	Magnetic Resonance Angiography (MRA)	
**3**	Noninvasive vascular lab (duplex, color flow, PVRs, etc.)	
**4**	Cardiac Imaging	
**5**	Arteriography (all: peripheral, renal mesenteric, carotid, etc.)	
**6**	Venography (all)	
**7**	Dialysis access evaluations	
**8**	Carotid artery imaging	
**9**	Venous access (all: tunneled, nontunneled, ports)	Vascular Intervention
**10**	IVC filter placement, retrieval	
**11**	Venous ablation (varicose veins)	
**12**	Dialysis access intervention	
**13**	TIPS & TIPS evaluation/revision	
**14**	Angioplasty/stents/covered stents: arterial (peripheral renal, mesenteric)	
**15**	Angioplasty/stents/covered stents: venous (all)	
**16**	Carotid stenting	
**17**	Thrombolytic therapy (all), thrombectomy	
**18**	Aortic endografting (thoracic and/or abdominal)	
**19**	Embolization, emergency (trauma, GI bleed, bronchial bleed, other)	
**20**	Embolization, elective (uterine fibroids, PAVMs, peripheral AVMs, varicoceles, etc.)	
**21**	Chemoembolization (TACE)	
**22**	Radioembolization (selective internal radiotherapy)	
**23**	Other, Vascular	
**24**	Biopsy	Non Vascular Intervention
**25**	Abscess drainage & tube checks	
**26**	Paracentesis, thoracentesis	
**27**	Chest tube placement	
**28**	Pleurodesis	
**29**	PTC, biliary drainage, biliary stents; tube checks	
**30**	Nephrostomy, nephroureterostomy; tube checks	
**31**	Gastrostomy, gastrojejunostomy; tube checks	
**32**	Cholecystostomy; tube checks	
**33**	Aspiration, drainage, sclerosis (cyst, lymphocele); tube checks	
**34**	Stents, miscellaneous nonvascular (esophageal, tracheobronchial, duodenal, colonic)	
**35**	Transplant interventions, miscellaneous	
**36**	Tumor ablation (RFA, laser, microwave, cryo, ethanol, other)	
**37**	Pain management	
**38**	Fallopian tube recanalization	
**39**	Other, Nonvascular	

#### Cost calculation for local model

We performed inference on an NVIDIA A100 GPU with 24GB VRAM equipped server. To quantify its operational cost, we considered three main components: hardware depreciation, power consumption, and recurring overhead (e.g., maintenance, physical space, and administrative costs). The A100 GPU with 24GB VRAM has an upfront cost of approximately $8,000 USD.^36^^,^^37^ Commercial entities typically amortize such hardware over three years but we adopt a more conservative five-year depreciation schedule to reflect longer usage cycles common in academic environments. This results in an hourly depreciation cost of roughly $0.23.

The estimated power draw of the server—including GPU, CPU, memory, storage, and cooling infrastructure—is approximately 400 watts.^38^ At an average electricity rate of $0.19 per kilowatt-hour^39^ (US average), this corresponds to $0.06 in energy costs per hour. To account for auxiliary operational expenses, we add a 20% overhead margin to the base cost, contributing an additional $0.06 per hour.

Summing these components, the total estimated cost of operating the server is $0.35 per hour. When expressed on a per-second basis, this translates to an operational cost of approximately $**0.000097**, or **0.01 cents per second**. This estimate serves as a realistic baseline for evaluating the cost-efficiency of locally hosted LLM inference in academic and research settings.

Table 3 presents the per-procedure and per-report cost of deploying the Claude-3.5-Haiku model in a commercial environment via AWS Bedrock. Unlike local deployments, commercial usage is priced based on token consumption—typically charged per input and output token. Thus, for the commercial model, costs were calculated based on the number of input and generated tokens per procedure under each prompting strategy.

#### Cost calculation for the commercial model

Unlike local deployments, commercial language models such as Claude-3.5-Haiku are billed based on token usage, with separate rates for input and output tokens. According to the official AWS Bedrock pricing documentation,^40^ the cost is $0.0008 per 1,000 input tokens and $0.004 per 1,000 output tokens.

Therefore, for a given inference task—such as identifying whether a procedure was performed—if the input prompt contains 1,000 tokens and the model generates a 1,000-token response, the total cost for that inference would be $0.0008+$0.004=$0.0048.

#### Comparative cost of local vs Commercial deployment

For the local model, see Table 3, with a per-second operating cost of $0.000097 (derived above), the estimated cost per 9.7s procedure is $0.00094 using IP and $0.00131 using CoT. Extrapolated to full reports containing an average of 39 procedures, the per-report costs are $0.03670 (IP) and $0.05096 (CoT)—affordable in many academic and clinical research settings.

On the commercial side, Table 3 shows IP prompts cost $0.0008 per procedure and $0.0324 per report, while CoT prompts cost slightly more ($0.0011 per procedure; $0.0433 per report), due to increased input token length. These values indicate that Claude’s commercial deployment is competitive with local deployment of Qwen, particularly when factoring in maintenance-free scalability, security compliance, and minimal hardware investment.

#### Comparison with manual logging burden

Radiology residents are responsible for documenting upwards of 1,600 reports during the time of residency, with each report averaging 39 distinct procedural entries. Assuming just 2 seconds per manual entry, this amounts to:


$$\begin{array}{cc} \text{Total}\;\text{manual}\;\text{annotation}\;\text{time} & =1,600\times 39\times 2\;\text{seconds}\\ & =124,800\;\text{seconds}\\ & \approx 35\;\text{hours}. \end{array}$$

This represents almost one full-time workweek spent on clerical documentation per resident—time that could be redirected toward patient care, educational development, or research. Furthermore, manual annotation is susceptible to human error, variability in documentation diligence, and inconsistent understanding of procedural terminology—all of which degrade data quality in high-stakes applications like credentialing and competency tracking.

## Discussion

### Limitations & future work

This study has several limitations that open avenues for future research.

First, the analysis was performed on a relatively small dataset comprising 414 radiology reports. This limited sample size, driven primarily by data availability and labeling time constraints, may affect the generalizability of the findings in broader clinical contexts. Future work should aim to validate these results using a larger and more diverse dataset spanning multiple institutions, imaging modalities, and patient demographics.

Second, our evaluation focused on a single commercially available model on the Bedrock platform Claude-3.5-Haiku (as of February, 2025). Although it demonstrated strong performance across prompting strategies, the commercial LLM landscape is evolving rapidly, with emerging models offering varying trade-offs in speed, cost, and reasoning capabilities. Notably, widely-used commercial models such as OpenAI’s GPT-4 and Google’s Gemini were not evaluated, as their API platforms were not available under our institutional HIPAA-compliant infrastructure at the time of the study; AWS Bedrock was the only platform covered under our institutional data use agreement that provided the necessary HIPAA-eligible guarantees. Future studies should conduct comparative evaluations across a wider range of HIPAA-compliant commercial platforms, including OpenAI’s Azure-hosted API and Google Cloud’s Vertex AI, to identify the most suitable models for specific clinical use cases and infrastructure constraints, and to strengthen the generalizability of these findings.

Third, we limited our experimentation to two prompting strategies: IP and CoT. Although these are among the most commonly adopted prompting paradigms, recent advances have introduced a variety of alternatives—including few-shot prompting, self-consistency prompting, and tool-augmented prompting; that may further enhance model performance, interpretability, or robustness. Incorporating and benchmarking these strategies could provide deeper insights into optimizing LLM-driven clinical automation.

Fourth, while this study focused on procedural extraction from radiology reports, the broader clinical documentation landscape includes many other entities, such as diagnoses, findings, measurements, and recommendations, that were not addressed here. Expanding the scope of LLM applications to include these additional dimensions could contribute to more comprehensive automation and structured documentation in healthcare workflows.

Finally, the evaluation was limited to a single institution with a specific reporting style and workflow. Radiology documentation conventions differ across hospitals, specialties, and even individual clinicians. As such, the reported performance may not generalize to other settings. Multi-institutional validation across varied EHR and PACS systems is essential before clinical deployment.

Future research should therefore pursue multi-site collaborations, larger annotated corpora, and expanded prompting paradigms. Equally important is assessing usability and trust among residents, faculty, and credentialing bodies, as automation bias or over-reliance on AI outputs could compromise oversight. Integrating LLM-based tools in a way that supports, rather than replaces, human review will be critical. By addressing these gaps, the field can move toward safe, equitable, and scalable automation of clinical documentation in medical education.

### Conclusion

This study evaluated the use of large language models to automate procedural case log documentation in radiology training. Both open-weight (Qwen-2.5:72B) and commercial (Claude-3.5-Haiku) models outperformed a metadata-only benchmark, reliably identifying common procedures while struggling with categories marked by vague or overlapping language. Operational analyses showed that local and commercial deployments offer broadly comparable costs, with trade-offs between speed, scalability, and data privacy. These findings suggest that LLMs are a feasible complement to current documentation workflows, offering the potential to reduce clerical burden, though further validation across institutions, procedures, and prompting strategies is required to ensure safe integration into training and clinical practice.

## Supplementary Material

ooag067_Supplementary_Data

## Data Availability

The project codebase and its documentation are available on a dedicated website, accessible here: https://nafiz43.github.io/PCL-Fetcher/. Due to confidentiality, the data has not been made available.
