# Supplementary material for: Intelligent documentation in medical education: can AI replace manual case logging?
Source: JAMIA Open. 2026 May 28;9(3):ooag067. doi: 10.1093/jamiaopen/ooag067 (PMC13221242; doi:10.1093/jamiaopen/ooag067)
Supplement: ooag067_Supplementary_Data [file ooag067_supplementary_data.pdf]

Appendix A. Prompt Templates

```
I will provide you with a radiology report, followed by a question about whether a specific radiology study or
↪  procedure was performed.

<procedure\_specific\_question>

### **Strict Output Format**
Your response **must** be a **valid JSON object** with the following keys:
```json
{
  "reason_for_the_label": "A concise explanation justifying the classification.",
  "label": 1 or 0
}
```

Listing 1: Prompt Template for Chain-of-Thought prompting, in which the <procedure_specific_question> placeholder is dynamically replaced with a specific question tailored to the target radiology procedure

```
I will provide you with a radiology report, followed by several questions about it. Your task is to determine
↪ whether a specific radiology study or procedure was performed. Please follow these strict formatting
↪ guidelines for your response:

Output must be in valid JSON format with the following keys:

{
  "reason_for_the_label": "A string explaining the reasoning behind the classification.",
  "label": 1 or 0
}

Labeling criteria:
Return 1 if the radiology study or procedure was explicitly mentioned as performed.
Return 0 if the study or procedure was not performed, not documented, or uncertain in the report.
Do not include any additional text or explanations outside the JSON response.
Ensure strict adherence to this format for every response
```

Listing 2: Prompt Template for Instruction Prompting

Appendix B. Server Configuration

We conducted all the experiments for our study on a dedicated local server with a well-defined configuration to ensure consistency and reliability in our results. The configuration of the server is presented in table B.1.

Appendix C. Performance & Cost of the Additional Local Models

The performance and cost metrics of the additional local models are presented in Table 2 and Table 3, respectively. Table 2 highlights their effectiveness across different tasks and prompting strategies, while Table 3 provides a breakdown of the associated computational costs

Appendix D. Model Error Count
```

Table B.1: Local server configuration

Component	Configuration
Primary Memory (RAM)	64 GB
Secondary Memory (Hard Disk)	512 GB SSD
Processor	*Name*: Intel(R) Xeon(R) W-2135 CPU @ 3.70GHz<br>*Architecture*: x86_64<br>*CPU(s)*: 12<br>*Thread(s) per core*: 2<br>*Core(s) per socket*: 6<br>*Max Clock Speed*: 4500.00 MHz<br>*Min Clock Speed*: 1200.00 MHz<br>*PCI Express Lanes*: 48<br>*Integrated Graphics*: None<br>*TDP (Thermal Design Power)*: 140 W
Cache	*L1d cache*: 192 KiB (6 instances)<br>*L1i cache*: 192 KiB (6 instances)<br>*L2 cache*: 6 MiB (6 instances)<br>*L3 cache*: 8.3 MiB (1 instance)
GPU	*Name*: A100 (PCIe)<br>*Memory*: 24 GB HBM2<br>*Memory Interface Width*: 3840 bits<br>*Memory Bandwidth*: 1555 GB/s<br>*Base Clock Speed*: 1095 MHz<br>*Boost Clock Speed*: 1410 MHz<br>*Memory Clock Speed*: 1215 MHz<br>*CUDA Cores*: 6912<br>*Tensor Cores*: 432 (3rd Gen)<br>*Thermal Design Power (TDP)*: 250 W

Appendix E. Description of the selected Models

`Mixtral-8x7B`. Mixtral-8x7B [1] is a mixture-of-experts model released by Mistral AI. The 8x7B version activates 2 out of 8 expert pathways per token, which offers a balance between model expressiveness and computational efficiency. It has shown competitive performance across a range of multilingual and general-purpose NLP tasks.

`LLaMA 3.3-70B`. LLaMA 3 is the latest generation of Meta's foundational language models. The 70B version demonstrates significant gains in general understanding, reasoning, and instruction-following [2]. It is particularly effective for downstream fine-tuning and multi-turn prompt applications.

`Qwen 2.5-72B`. Qwen 2.5 [3] is a high-performance transformer model developed by Alibaba. The 72B version is instruction-tuned and optimized for accuracy in information extraction tasks. It supports a wide multilingual range and is designed for competitive performance in both zero- and few-shot settings.

`MedLLaMA-2-7B`. MedLLaMA-2-7B is a biomedical adaptation of Meta's LLaMA architecture, fine-tuned on large-scale medical corpora. The 7B parameter variant focuses on healthcare and clinical domains, making it suitable for domain-specific tasks such as radiology procedural report interpretation.

`LLaMA-3-Med42-70B`. LLaMA-3-Med42-70B [4] is a domain-specialized version of LLaMA 3.3-70B, fine-tuned on a curated medical dataset named Med42 [**?**]. It is intended for complex clinical reasoning, procedural understanding, and structured medical data generation.

`Claude-3.5-Haiku`. Claude 3.5 Haiku [5] is a lightweight commercial LLM developed by Anthropic. Despite its smaller size, it exhibits strong instruction-following and chain-of-thought reasoning capabilities. It is optimized for fast response times while maintaining high accuracy in structured generation tasks.

[1] https://huggingface.co/mistralai/Mixtral-8x7B-v0.1
[2] https://huggingface.co/meta-llama/Llama-3.3-70B-Instruct
[3] https://qwenlm.github.io/blog/qwen2.5/
[4] https://huggingface.co/m42-health/Llama3-Med42-70B
[5] https://www.anthropic.com/claude/haiku

Table C.2: Performance of the Crosswalk Benchmark Compared to the Top-Performing Local and Commercial Language Model

Model-Name	Prompting Method	Modality	TP	TN	FP	FN	Sensitivity (%)	Specificity (%)	F1-Score (%)
Llama3-Med42:70B	IP	All	662	14714	743	27	96.08	95.19	63.23
	IP	VascularDiagnosis	215	3039	49	9	95.98	98.41	88.11
	IP	VascularIntervention	257	5559	385	9	96.62	93.52	56.61
	IP	NonVascularIntervention	190	6116	309	9	95.48	95.19	54.45
	CoT	All	645	15037	420	44	93.61	97.28	73.54
	CoT	VascularDiagnosis	211	3042	46	13	94.20	98.51	87.73
	CoT	VascularIntervention	250	5816	128	16	93.98	97.85	77.64
	CoT	NonVascularIntervention	184	6179	246	15	92.46	96.17	58.50
Llama3.3:70B	IP	All	662	14826	631	27	96.08	95.92	66.80
	IP	VascularDiagnosis	215	3048	40	9	95.98	98.70	89.77
	IP	VascularIntervention	256	5647	297	10	96.24	95.00	62.51
	IP	NonVascularIntervention	191	6131	294	8	95.98	95.42	55.85
	CoT	All	628	15191	266	61	91.15	98.28	79.35
	CoT	VascularDiagnosis	200	3052	36	24	89.29	98.83	86.96
	CoT	VascularIntervention	249	5834	110	17	93.61	98.15	79.68
	CoT	NonVascularIntervention	179	6305	120	20	89.95	98.13	71.89
Medllama2:7B	IP	All	321	8909	6548	368	46.59	57.64	8.49
	IP	VascularDiagnosis	126	1959	1129	98	56.25	63.44	17.04
	IP	VascularIntervention	107	3372	2572	159	40.23	56.73	7.26
	IP	NonVascularIntervention	88	3578	2847	111	44.22	55.69	5.62
	CoT	All	605	3771	11686	84	87.81	24.40	9.32
	CoT	VascularDiagnosis	206	885	2203	18	91.96	28.66	15.65
	CoT	VascularIntervention	245	412	5532	21	92.11	6.93	8.11
	CoT	NonVascularIntervention	154	2474	3951	45	77.39	38.51	7.15
Mixtral:8x7B	IP	All	623	14728	729	66	90.42	95.28	61.05
	IP	VascularDiagnosis	194	3050	38	30	86.61	98.77	85.09
	IP	VascularIntervention	243	5608	336	23	91.35	94.35	57.52
	IP	NonVascularIntervention	186	6070	355	13	93.47	94.47	50.27
	CoT	All	601	14444	1013	88	87.23	93.45	52.20
	CoT	VascularDiagnosis	199	2940	148	25	88.84	95.21	69.70
	CoT	VascularIntervention	234	5500	444	32	87.97	92.53	49.57
	CoT	NonVascularIntervention	168	6004	421	31	84.42	93.45	42.64

Table C.3: Cost of Running the Language Model in Relation to Inference Time and Generated Tokens for the Rest of the Local Models

Model Name	Prompting Method	Inference Time (Second)				Generated Tokens (Count)			
		Max	Min	Mean	Std Dev	Max	Min	Mean	Std Dev
Llama3-Med42:70B	IP	13.00	3.00	6.53	1.65	147	14	53.81	17.92
	CoT	18.00	7.00	10.44	1.80	152	34	70.65	16.61
Llama3:70B	IP	330.00	2.00	57.32	21.66	433	1	71.03	29.69
	CoT	59.00	11.00	28.52	4.67	84	12	29.10	10.28
Medllama2:7B	IP	1497.00	0.00	2.97	47.93	66420	13	135.31	2125.40
	CoT	1027.00	0.00	3.83	56.79	74564	5	244.15	4004.62
Mixtral:8x7B	IP	32.00	0.00	0.98	1.17	116	1	30.20	17.07
	CoT	9.00	1.00	2.21	0.77	124	14	41.83	16.65

Table D.4: Classification error count (False Postive and False Negative) of the models on each of the procedures

**Procedure Number**	**Crosswalk**		**Claude-3.5-Haiku**		**Qwen-2.5:72B**	
	**FP Count**	**FN Count**	**FP Count**	**FN Count**	**FP Count**	**FN Count**
1	1	21	8	0	10	0
2	0	0	1	0	0	0
3	0	9	5	0	3	0
4	10	1	0	0	0	0
5	29	77	1	2	1	3
6	22	57	2	10	1	6
7	0	20	1	2	1	0
8	0	39	1	0	1	1
9	0	13	2	4	2	6
10	1	8	1	0	0	0
11	0	0	3	0	2	0
12	0	8	1	1	0	1
13	6	3	0	1	0	1
14	0	12	4	0	3	0
15	0	74	3	2	5	1
16	0	3	1	0	1	0
17	15	36	2	0	2	0
18	0	0	0	0	0	0
19	30	62	3	14	1	9
20	39	56	7	4	15	2
21	0	3	1	1	0	1
22	3	0	0	2	0	2
23	6	24	11	9	45	1
24	29	2	3	1	1	1
25	14	14	5	1	1	3
26	3	13	4	0	6	0
27	0	4	0	0	0	0
28	0	0	1	0	0	0
29	7	8	1	1	0	1
30	0	5	1	1	0	1
31	0	3	5	0	3	0
32	5	14	1	0	0	0
33	4	4	2	1	1	1
34	0	3	7	1	14	1
35	0	9	6	0	5	0
36	0	0	0	0	0	0
37	0	6	0	5	0	6
38	0	0	0	0	0	0
39	20	41	15	13	7	14
**Total**	**244**	**652**	**109**	**76**	**131**	**62**

Appendix F. LLM Prompts

This section outlines the various prompting techniques employed in the study.

Appendix F.1. Instruction Prompting

`Prompt for Question 1:`

```
Please answer 1 if the report explicitly states that a CTA (CT Angiography) or an intra-operative cone-beam CT
↪  with contrast was performed. If a CTA or intra-operative cone-beam CT with contrast was not performed or
↪ not mentioned, please answer 0.
```

`Prompt for Question 2:`

```
Please answer 1 if the report specifies an MRA or MR Angiography was performed. Otherwise, please answer 0
```

`Prompt for Question 3:`

```
Please answer 1 if the report specifies a noninvasive vascular lab study was performed. Examples include a
↪ duplex, color flow, or Pulse Volume Recording (PVR). Otherwise, please answer 0
```

`Prompt for Question 4:`

```
Please answer 1 if the report specifies a cardiac imaging study was performed. Examples include a CT coronary
↪ angiogram or nuclear medicine cardiac stress test. Otherwise, please answer 0
```

`Prompt for Question 5:`

```
Please answer 1 if a dedicated arteriography was performed. Arteriography is defined as imaging of arteries
↪ using fluoroscopy and contrast specifically to evaluate arterial anatomy or pathology. The contrast must be
↪  specifically administered into an artery. Arteriography does not include venography. Answr 0 if
↪ arteriography was not performed or not documented
```

`Prompt for Question 6:`

```
Please answer 1 if a dedicated venography was performed . Venorgaphy is defined as imaging of veins using
↪ fluoroscopy and contrast specifically to evaluate venous anatomy or pathology. The contrast must be
↪ specifically administered into a vein. Venography does not include arteriography. Answer 0 if no dedicated
↪ venography was performed
```

`Prompt for Question 7:`

```
Please answer 1 if a dialysis access evaluation was performed. These evaluations include ultrasound studies,
↪ fistulagrams, and graft evaluations. Answer 0 if a dialysis access evaluation was not performed or not
↪ documented
```

`Prompt for Question 8:`

```
Please answer 1 if carotid artery imaging was performed. Answer 0 if carotid artery imaging was not performed
↪ or not documented
```

`Prompt for Question 9:`

```
Please answer 1 if a central venous catheter procedure was performed. This includes placement, removal, and
↪ revision of central venous catheters, ports, and PICCs. Answer 0 if a central venous catheter procedure was
↪  not performed or not documented
```

`Prompt for Question 10:`

```
Please answer 1 if an IVC filter placement or removal procedure was performed. Answer 0 if an IVC filter
↪ procedure was not performed or not documented
```

`Prompt for Question 11:`

```
Please answer 1 if a venous ablation procedure was performed. Venous ablations are performed for varicose
↪ veins,. Answer 0 if venous ablation was not performed or not documented
```

`Prompt for Question 12:`

```
Please answer 1 if a dialysis access intervention was performed. These interventions inlcude fistulagrams,
↪ graft evaluations, and declot procedures specifically and only on arteriovenous access. Answer 0 if a
↪ dialysis access intervention was not performed or not documented
```

Prompt for Question 13:

```
Please answer 1 if any intervention involving a portosystemic shunt was performed. Typical shunts include
↪ Transjugular Intrahepatic Portosystemic Shunt (TIPS), Direct intrahepatic portocaval shunt (DIPS) and
↪ transjugular transcaval intrahepatic portosystemic shunt (TTIPS). Interventions inlcude shunt placement,
↪ evaluation, or revision. Answer 0 if a portosystemic shunt intervention was not performed or not documented
```

Prompt for Question 14:

```
Please answer 1 if angioplasty or stent placement in the arterial system was performed. The arterial system is
↪  defined as any artery in the body. Answer 0 if angioplasty or stent placement in the arterial system was
↪ not performed or not documented
```

Prompt for Question 15:

```
Please answer 1 if angioplasty or stent placement in the venous system was performed. The venous system is
↪ defined as any vein in the body. Angioplasty means any inflation of a balloon in the venous system for
↪ opening a lumen or for mechanical disruption. Answer 0 if angioplasty or stent placement in the venous
↪ system was not performed or not documented
```

Prompt for Question 16:

```
Please answer 1 if stent placement in the carotid artery was performed and 0 if it was not performed or not
↪ documented
```

Prompt for Question 17:

```
Please answer 1 if thrombolytic therapy or thrombectomy was performed. Thrombolytic therapy may be
↪ administration of any tissue plasminogen activator (tPA) usch as tenecteplase or alteplase. Thrombectomy
↪ may include mechanical suction thrombectomy, balloon thrombectomy, or pharmacomechanical thrombectomy.
↪ Answer 0 if thrombolytic therapy or thrombectomy was not performed or not documented
```

Prompt for Question 18:

```
Please answer 1 if aortic endograft placement or revision was performed and 0 if it was not performed or not
↪ documented
```

Prompt for Question 19:

```
Please answer 1 if an emergency embolization was performed. Emergency embolization is defined as the
↪ intravascular administration of an occlusive material to stop or control bleeding and hemrrohage. Possible
↪ indications include trauma, gastrointestinal (GI) bleeding, hemoptysis, iatrogenic, and tumoral.
↪ Embolization materials may include Gelfoam, Onyx, beads, coils, and plugs. Answer 0 if an emergency
↪ embolization was not performed or not documented
```

Prompt for Question 20:

```
Please answer 1 if an elective embolization was performed. Elective embolization is defined as the
↪ intravascular administration of an occlusive material to stop or reduce flow of blood or for administration
↪  of a medication. Elective embolization procedures include uterine artery emboliztion (UAE), uterine
↪ fibroid embolization (UFE), arteriovenous malformation (AVM) embolization, varicocele embolization, and
↪ gondal vein embolization. Transarterial chemoembolization (TACE) and transarterial radioemboliztion (TARE)
↪ should not be included in this group unless protective embolization was performed. Answer 0 if an elective
↪ embolization was not performed or not documented
```

Prompt for Question 21:

```
Please answer 1 if a transarterial chemoembolization (TACE) was performed. TACE is defined as the
↪ intravascular administration of a chemotherapy gent to treat a tumor. Answer 0 if a TACE procedure was not
↪ performed or not documented
```

Prompt for Question 22:

```
Please answer 1 if a transarterial radioembolization (TARE) procedure was performed. TARE procedures include
↪ angiographic mapping with or without MAA administration and yttrium-90 (Y90) administration. Answer 0 if a
↪ TARE procedure was not performed or not documented
```

Prompt for Question 23:

```
Please answer 1 if an intravascular procedure was performed that doesn't fit into one of the following
↪ categories: Venous access (such as port placement, exchange, or removal, or central venous catheter
↪ placement, exchange, or removal, or dialysis catheter placement, exchange, or removal), dialysis access
↪ intervention, IVC filter placement or removal, ablation, stent or stent-graft placement, TIPS, DIPS,
↪ embolization, TACE, or TARE. Examples might include a transjugular liver or renal biopsy, other
↪ intravscalar biopsy or fiducial placement. Otherwise, please answer 0
```

Prompt for Question 24:

```
Please answer 1 if a percutaneous biopsy was performed and 0 if it was not performed or not documented.
↪ Percutaneous biopsy does not include intravascular biopsy procedures such as transjugular liver biopsy and
↪ transsjugular renal biopsy
```

Prompt for Question 25:

```
Please answer 1 if an abscess drainage procedure was performed. These procedures include an abscess or fluid
↪ collection drain placement, revision, repositioning, upsize, or removal. This excludes biliary drains and
↪ tubes, cholecystostomy drains and tubes, nephrostomy drains and tubes, nephroureterostomy drains and tubes,
↪  and chest drains and tubes. Answer 0 if an abscess drainage procedure was not performed or not documented
```

Prompt for Question 26:

```
Please answer 1 if  paracentesis or thoracentesis was performed and 0 if it was not performed or not
↪ documented
```

Prompt for Question 27:

```
Please answer 1 if a chest tube placement was performed and 0 if it was not performed or not documented
```

Prompt for Question 28:

```
Please answer 1 if pleurodesis was performed and 0 if it was not performed or not documented
```

Prompt for Question 29:

```
Please answer 1 if any procedure involving a biliary drain or stent was performed. These procedures include
↪ percutaneous transhepatic cholangiography (PTC), biliary drain placement, evaluation, or revision, and
↪ biliary stent placement or revision. Cholecystostomy placement/exchange/removal procedures are not
↪ considered a biliary drain or stent procedure. Answer 0 if no biliary drain or biliary stent procedure was
↪ performed or documented
```

Prompt for Question 30:

```
Please answer 1 if any procedure involving a geniturinary drain procedure was performed. These procedures
↪ include nephrostomy tube and nephrourteral stent placement, exchange, revision, or removal. Answer 0 if any
↪  procedure involving a geniturinary drain procedure was not performed or not documented
```

Prompt for Question 31:

```
Please answer 1 if any enterostomy procedure was performed. These procedures include gastrostomy,
↪ gastrojejunostomy, jejunostromy, iliostomy, and cecostomy placement, exchange, revision, or removal. Answer
↪  0 if an enterostomy procedure was not performed or not documented
```

Prompt for Question 32:

```
Please answer 1 if any cholecystostomy procedure was performed. These procedures include cholecystostomy drain
↪  or tube placement, exchange, revision, or removal and cholecystography. Answer 0 if a cholecystostomy
↪ procedure was not performed or not documented
```

Prompt for Question 33:

```
Please answer 1 if a cyst or lymphocele procedure was performed. These procedures include aspiration, drain
↪ placement, exchange, revision, or removal and sclerosis of a cyst or lymphocele. This does not include
↪ interventions on abscesses or other fluid collections. Answer 0 if a cyst or lymphocele procedure was not
↪ performed or not documented
```

Prompt for Question 34:

```
Please answer 1 if a nonvascular stent placement was performed. Nonvascular stents include esophageal,
↪ tracheobronchial, duodenal, and colonic stents. Answer 0 if a nonvascular stent placement was not performed
↪  or not documented
```

Prompt for Question 35:

```
Please answer 1 if a miscellaneous nonvascular transplant intervention was performed. Miscellaneous
↪ nonvascular transplant interventions are interventions performed exclusively on a transplant organ outside
↪ of the arteries and veins. The text must explicitly report that the organ is a transplant organ. A
↪ transplant organ is not a native organ. Intravascular procedures involving a transplant organ, such as
↪ transplant renal artery stenosis (TRAS) or transjular biopsy, should not considered as examples because
↪ they are vascular. Examples of nonvascular transplant interventions include stent placements involving a
↪ transplant organ or balloon plasty procedures such as ureteroplasty. Procedures such as nephrostomy and
↪ nephroureterostomy placement, exchange, or removal and biliary tube placement, exchange, and removal should
↪  NOT be considered in this miscellaneous category. Answer 0 if a miscellaneous nonvascular transplant
↪ intervention was not performed or not documented
```

Prompt for Question 36:

```
Please answer 1 if a tumor ablation was performed. Tumor ablations may include radiofrequency (RFA), laser,
↪ microwave, cryoablation, and ethanol administration. Answer 0 if a tumor ablation was not performed or not
↪ documented
```

Prompt for Question 37:

```
Please answer 1 if a pain managment procedure was performed. Pain managment procedures may include steroid
↪ injection, celiac plexus neurolysis, or nerve and periosteal cryoablation. Answer 0 if a pain managment
↪ procedure was not performed or not documented
```

Prompt for Question 38:

```
Please answer 1 if a Fallopian tube recanalization was performed and 0 if it was not performed or not
↪ documented
```

Prompt for Question 39:

```
An invasive procedure involves inserting instruments or devices into the body, typically through the skin or a
↪  natural body orifice. Please answer 1 if the report specifies that a nonvascular, invasive procedure was
↪ performed and it does not fall into any of the following categories: Biopsy, Abscess drainage & tube checks
↪ , Paracentesis, Thoracentesis, Chest tube placement, Pleurodesis, PTC, Biliary drainage catheter placement/
↪ exchange/removal, Biliary stent placement/exchange/removal, Tube checks, Genitourinary catheter placement/
↪ exchange/removal, Nephrostomy placement/exchange/removal, Nephroureteral tube placement/exchange/removal,
↪ Gastrostomy placement/exchange/removal, Gastrojejunostomy placement/exchange/removal, Cholecystostomy
↪ placement/exchange/removal, Cyst or lymphocele intervention, Nonvascular stents (esophageal,
↪ tracheobronchial, duodenal, colonic), Miscellaneous transplant interventions, Tumor ablation, Pain
↪ management interventions, Fallopian tube recanalization, or any intravascular procedure (including venous
↪ access procedures such as port placement, exchange, or removal; central venous catheter placement, exchange
↪ , or removal; dialysis catheter placement, exchange, or removal; dialysis access intervention; IVC filter
↪ placement or removal; Ablation; Stent or stent-graft placement; TIPS; DIPS; Embolization; Transarterial
↪ chemoembolization (TACE); or Transarterial radioembolization (TARE)). If a non-vascuar invasive procedure
↪ was not performed, please answer 0.
```

Appendix F.2. Chain-of-Thought

Prompt for Question 1:

```
Your task is to determine whether a **CTA (CT Angiography)** or **intra-operative cone-beam CT with contrast** was explicitly performed.

If the procedure was explicitly performed, return ‘1‘ along with an explanation. Otherwise, return ‘0‘ with an explanation.

Follow a structured reasoning approach to do the task:

### **Reasoning Steps**
1. **Identify Mentions**: Extract all references to CTA or intra-operative cone-beam CT with contrast in the report.
2. **Assess Explicitness**: Determine if the report explicitly states that the procedure **was performed** (e.g., ‘CTA was conducted,’ ’A cone-beam CT with contrast was completed’).
3. **Resolve Ambiguities**: If the procedure is only **suggested, planned, considered, or recommended**, but there is no explicit confirmation that it was performed, classify it as **not performed**.
4. **Handle Uncertainty**: If the report lacks a direct mention of the procedure, assume it was **not performed**.
5. **Generate JSON Output**: Construct a response in strict JSON format, adhering to the following structure:
```

Prompt for Question 2:

```
Your task is to determine whether an **MRA (MR Angiography)** was explicitly performed.

If the procedure was explicitly performed, return ‘1‘ along with an explanation. Otherwise, return ‘0‘ with an explanation.

Follow a structured reasoning approach to do the task:

### **Reasoning Steps**
1. **Identify Mentions**: Extract all references to MRA (MR Angiography) in the report.
2. **Assess Explicitness**: Determine if the report explicitly states that the procedure **was performed** (e.g., ’MRA was conducted,’ ’MR Angiography was completed’).
3. **Resolve Ambiguities**: If the procedure is only **suggested, planned, considered, or recommended**, but there is no explicit confirmation that it was performed, classify it as **not performed**.
4. **Handle Uncertainty**: If the report lacks a direct mention of MRA, assume it was **not performed**.
5. **Generate JSON Output**: Construct a response in strict JSON format, adhering to the following structure:
```

Prompt for Question 3:

```
Your task is to determine whether a **noninvasive vascular lab study** was explicitly performed. Examples include **duplex ultrasound, color flow study, or Pulse Volume Recording (PVR)**.

If the procedure was explicitly performed, return ‘1‘ along with an explanation. Otherwise, return ‘0‘ with an explanation.

Follow a structured reasoning approach to do the task:

### **Reasoning Steps**
1. **Identify Mentions**: Extract all references to **duplex ultrasound, color flow study, Pulse Volume Recording (PVR), or any other noninvasive vascular lab study** in the report.
2. **Assess Explicitness**: Determine if the report clearly states that the procedure **was performed** (e.g., ’A duplex ultrasound was conducted,’ ’PVR testing was completed’).
3. **Resolve Ambiguities**: If the procedure is only **suggested, planned, considered, or recommended** but not explicitly confirmed as performed, classify it as **not performed**.
4. **Handle Uncertainty**: If the report lacks a direct mention of a noninvasive vascular lab study, assume it **was not performed**.
5. **Generate JSON Output**: Construct a response in strict JSON format, adhering to the following structure:
```

Prompt for Question 4:

```
Your task is to determine whether a **cardiac imaging study** was explicitly performed. Examples include a ** CT coronary angiogram** or **nuclear medicine cardiac stress test**.

If the procedure was explicitly performed, return `1` along with an explanation. Otherwise, return `0` with an explanation.

Follow a structured reasoning approach to do the task:

### **Reasoning Steps**
1. **Identify Mentions**: Extract all references to **CT coronary angiogram, nuclear medicine cardiac stress test, or any other cardiac imaging study** in the report.
2. **Assess Explicitness**: Determine if the report clearly states that the procedure **was performed** (e.g., 'A CT coronary angiogram was conducted,' 'A nuclear medicine cardiac stress test was completed').
3. **Resolve Ambiguities**: If the procedure is only **suggested, planned, considered, or recommended** but not explicitly confirmed as performed, classify it as **not performed**.
4. **Handle Uncertainty**: If the report lacks a direct mention of a cardiac imaging study, assume it **was not performed**.
5. **Generate JSON Output**: Construct a response in strict JSON format, adhering to the following structure:
```

Prompt for Question 5:

```
Your task is to determine whether a **dedicated arteriography** was explicitly performed. **Arteriography** is defined as imaging of arteries using **fluoroscopy and contrast** specifically to evaluate arterial anatomy or pathology. The contrast **must be administered directly into an artery**. **Venography does not count as arteriography.**

If the procedure was explicitly performed, return `1` along with an explanation. Otherwise, return `0` with an explanation.

Follow a structured reasoning approach to do the task:

### **Reasoning Steps**
1. **Identify Mentions**: Extract all references to **arteriography** in the report. Look for terms like ** arteriogram, fluoroscopic arterial imaging, or direct intra-arterial contrast administration**.
2. **Confirm Modality & Contrast Administration**: Ensure the report specifies that the imaging was performed **using fluoroscopy and contrast**. The contrast **must** be administered **into an artery** to qualify as arteriography.
3. **Differentiate from Venography**: If the procedure involves **venous imaging** (e.g., venography or venogram), **do not classify it as arteriography**.
4. **Resolve Ambiguities**: If the procedure is only **suggested, planned, considered, or recommended** but not explicitly confirmed as performed, classify it as **not performed**.
5. **Handle Uncertainty**: If the report lacks a direct mention of **arteriography with intra-arterial contrast administration**, assume it **was not performed**.
6. **Generate JSON Output**: Construct a response in strict JSON format, adhering to the following structure:
```

Prompt for Question 6:

```
Your task is to determine whether a **dedicated venography** was explicitly performed. **Venography** is defined as imaging of veins using **fluoroscopy and contrast** specifically to evaluate **venous anatomy or pathology**. The contrast **must be administered directly into a vein**. **Arteriography does not count as venography.**

If the procedure was explicitly performed, return `1` along with an explanation. Otherwise, return `0` with an explanation.

Follow a structured reasoning approach to do the task:

### **Reasoning Steps**
1. **Identify Mentions**: Extract all references to **venography** in the report. Look for terms like ** venogram, fluoroscopic venous imaging, or direct intra-venous contrast administration**.
```

```
2. **Confirm Modality & Contrast Administration**: Ensure the report specifies that the imaging was performed **using fluoroscopy and contrast**. The contrast **must** be administered **into a vein** to qualify as venography.
3. **Differentiate from Arteriography**: If the procedure involves **arterial imaging** (e.g., arteriography or arteriogram), **do not classify it as venography**.
4. **Resolve Ambiguities**: If the procedure is only **suggested, planned, considered, or recommended** but not explicitly confirmed as performed, classify it as **not performed**.
5. **Handle Uncertainty**: If the report lacks a direct mention of a **venography with intra-venous contrast administration**, assume it **was not performed**.
6. **Generate JSON Output**: Construct a response in strict JSON format, adhering to the following structure:
```

Prompt for Question 7:

```
Your task is to determine whether a **dialysis access evaluation** was explicitly performed. These evaluations include:
- **Ultrasound studies** (e.g., Doppler ultrasound of dialysis access).
- **Fistulagrams** (fluoroscopic imaging of a dialysis fistula using contrast).
- **Graft evaluations** (imaging studies assessing the function and patency of dialysis grafts).

If the procedure was explicitly performed, return ‘1‘ along with an explanation. Otherwise, return ‘0‘ with an explanation.

Follow a structured reasoning approach to do the task:

### **Reasoning Steps**
1. **Identify Mentions**: Extract all references to **dialysis access evaluation** in the report. Look for terms such as **dialysis access ultrasound, fistulagram, or graft evaluation**.
2. **Confirm the Type of Study**: Ensure that the report explicitly states that one of the following procedures was performed:
   - **Ultrasound study** for dialysis access.
   - **Fistulagram** (contrast-based evaluation of a fistula).
   - **Graft evaluation** (assessment of a dialysis graft using imaging).
3. **Resolve Ambiguities**: If the procedure is only **suggested, planned, considered, or recommended** but not explicitly confirmed as performed, classify it as **not performed**.
4. **Handle Uncertainty**: If the report lacks a direct mention of any of the qualifying dialysis access studies, assume it **was not performed**.
5. **Generate JSON Output**: Construct a response in strict JSON format, adhering to the following structure:
```

Prompt for Question 8:

```
Your task is to determine whether **carotid artery imaging** was explicitly performed. This includes imaging studies specifically evaluating the carotid arteries, such as:
- **Carotid ultrasound (Doppler or duplex ultrasound of the carotid arteries)**
- **Carotid CT angiography (CTA)**
- **Carotid MR angiography (MRA)**
- **Carotid angiography (fluoroscopy with contrast administered into the carotid arteries)**

If the procedure was explicitly performed, return ‘1‘ along with an explanation. Otherwise, return ‘0‘ with an explanation.

Follow a structured reasoning approach to do the task:

### **Reasoning Steps**
1. **Identify Mentions**: Extract all references to **carotid artery imaging** in the report. Look for terms such as **carotid ultrasound, carotid Doppler, carotid CTA, carotid MRA, or carotid angiogram**.
2. **Confirm the Imaging Modality**: Ensure that the report explicitly states that one of the following procedures was performed:
   - **Ultrasound study** (e.g., Doppler or duplex of the carotid arteries).
   - **CT angiography (CTA)** of the carotid arteries.
   - **MR angiography (MRA)** of the carotid arteries.
   - **Catheter-based carotid angiography** (contrast-enhanced fluoroscopic evaluation).
```

```
3. **Resolve Ambiguities**: If the procedure is only **suggested, planned, considered, or recommended** but
↪ not explicitly confirmed as performed, classify it as **not performed**.
4. **Handle Uncertainty**: If the report lacks a direct mention of any carotid artery imaging study, assume it
↪  **was not performed**.
5. **Generate JSON Output**: Construct a response in strict JSON format, adhering to the following structure:
```

Prompt for Question 9:

```
Your task is to determine whether a **central venous catheter (CVC) procedure** was explicitly performed. This
↪  includes:
- **Placement** of a central venous catheter, port, or peripherally inserted central catheter (PICC).
- **Removal** of a central venous catheter, port, or PICC.
- **Revision** or adjustment of an existing central venous catheter, port, or PICC.

If the procedure was explicitly performed, return ‘1‘ along with an explanation. Otherwise, return ‘0‘ with an
↪  explanation.

Follow a structured reasoning approach to do the task:

### **Reasoning Steps**
1. **Identify Mentions**: Extract all references to **central venous catheter procedures** in the report. Look
↪  for terms such as **CVC placement, central line insertion, port-a-cath placement, PICC line insertion,
↪ catheter revision, or catheter removal**.
2. **Confirm Procedure Type**: Ensure that the report explicitly states that one of the following procedures
↪ was performed:
   - **Placement of a CVC, port, or PICC** (e.g., via ultrasound or fluoroscopic guidance).
   - **Removal of a CVC, port, or PICC** (e.g., catheter extraction or explantation).
   - **Revision or adjustment** of an existing CVC, port, or PICC.
3. **Resolve Ambiguities**: If the procedure is only **suggested, planned, considered, or recommended** but
↪ not explicitly confirmed as performed, classify it as **not performed**.
4. **Handle Uncertainty**: If the report lacks a direct mention of any **CVC-related procedure**, assume it **
↪ was not performed**.
5. **Generate JSON Output**: Construct a response in strict JSON format, adhering to the following structure:
```

Prompt for Question 10:

```
Your task is to determine whether an **Inferior Vena Cava (IVC) filter placement or removal procedure** was
↪ explicitly performed. This includes:
- **Placement** of an IVC filter (implantation of a filter within the inferior vena cava to prevent embolism).
- **Removal** or **retrieval** of an IVC filter (extraction of a previously placed filter).

If the procedure was explicitly performed, return ‘1‘ along with an explanation. Otherwise, return ‘0‘ with an
↪  explanation.

Follow a structured reasoning approach to do the task:

### **Reasoning Steps**
1. **Identify Mentions**: Extract all references to **IVC filter procedures** in the report. Look for terms
↪ such as **IVC filter placement, IVC filter removal, IVC filter retrieval, or caval filter implantation**.
2. **Confirm Procedure Type**: Ensure that the report explicitly states that one of the following procedures
↪ was performed:
   - **Placement of an IVC filter** (e.g., via catheter-based insertion in the inferior vena cava).
   - **Removal or retrieval of an existing IVC filter** (e.g., endovascular retrieval of the device).
3. **Resolve Ambiguities**: If the procedure is only **suggested, planned, considered, or recommended** but
↪ not explicitly confirmed as performed, classify it as **not performed**.
4. **Handle Uncertainty**: If the report lacks a direct mention of any **IVC filter placement or removal**,
↪ assume it **was not performed**.
5. **Generate JSON Output**: Construct a response in strict JSON format, adhering to the following structure:
```

Prompt for Question 11:

```
Your task is to determine whether a **venous ablation procedure** was explicitly performed. Venous ablations
↪ are performed primarily to treat **varicose veins** and involve techniques such as:
- **Endovenous laser ablation (EVLA)**
- **Radiofrequency ablation (RFA)**
- **Chemical sclerotherapy (foam or liquid injection for vein closure)**
- **Mechanochemical ablation (MOCA, ClariVein, etc.)**

If the procedure was explicitly performed, return `1` along with an explanation. Otherwise, return `0` with an
↪  explanation.

Follow a structured reasoning approach to do the task:

### **Reasoning Steps**
1. **Identify Mentions**: Extract all references to **venous ablation procedures** in the report. Look for
↪ terms such as **venous ablation, endovenous laser ablation (EVLA), radiofrequency ablation (RFA),
↪ sclerotherapy, or mechanochemical ablation**.
2. **Confirm the Procedure Type**: Ensure that the report explicitly states that one of the following
↪ procedures was performed:
   - **Endovenous thermal ablation (laser or radiofrequency-based techniques).**
   - **Sclerotherapy (chemical injection for vein closure).**
   - **Mechanochemical ablation (combining mechanical and chemical vein closure techniques).**
3. **Resolve Ambiguities**: If the procedure is only **suggested, planned, considered, or recommended** but
↪ not explicitly confirmed as performed, classify it as **not performed**.
4. **Handle Uncertainty**: If the report lacks a direct mention of any **venous ablation procedure**, assume
↪ it **was not performed**.
5. **Generate JSON Output**: Construct a response in strict JSON format, adhering to the following structure:
```

Prompt for Question 12:

```
Your task is to determine whether a **dialysis access intervention** was explicitly performed. These
↪ interventions are specific to **arteriovenous (AV) access** and include:
- **Fistulagrams** (angiographic evaluation of an AV fistula).
- **Graft evaluations** (imaging or interventional assessment of AV grafts).
- **Declot procedures** (thrombectomy or thrombolysis specifically performed on AV access).

If the procedure was explicitly performed, return `1` along with an explanation. Otherwise, return `0` with an
↪  explanation.

Follow a structured reasoning approach to do the task:

### **Reasoning Steps**
1. **Identify Mentions**: Extract all references to **dialysis access interventions** in the report. Look for
↪ terms such as **fistulagram, AV fistula angiography, AV graft evaluation, thrombectomy, thrombolysis, or
↪ declot procedure on an AV access**.
2. **Confirm the Procedure Type**: Ensure that the report explicitly states that one of the following
↪ procedures was performed **on an arteriovenous access**:
   - **Fistulagram** (AV fistula imaging and assessment).
   - **Graft evaluation** (assessment of an AV graft for function or complications).
   - **Declot procedure** (thrombectomy or thrombolysis **specifically on an AV access**).
3. **Resolve Ambiguities**: If the procedure is only **suggested, planned, considered, or recommended** but
↪ not explicitly confirmed as performed, classify it as **not performed**.
4. **Exclude Non-Relevant Procedures**: Ensure that procedures **not related to arteriovenous access** (such
↪ as central venous catheter interventions) **are not mistakenly classified as dialysis access interventions
↪ **.
5. **Handle Uncertainty**: If the report lacks a direct mention any **dialysis access intervention**, assume
↪ it **was not performed**.
6. **Generate JSON Output**: Construct a response in strict JSON format, adhering to the following structure:
```

Prompt for Question 13:

```
Your task is to determine whether **any intervention involving a portosystemic shunt** was explicitly performed. These interventions include:
- **Shunt placement** (creation of a new portosystemic shunt).
- **Shunt evaluation** (imaging or assessment of an existing shunt for patency or function).
- **Shunt revision** (modification, balloon angioplasty, or stenting of a pre-existing shunt).

The **portosystemic shunts** of interest include:
- **Transjugular Intrahepatic Portosystemic Shunt (TIPS)**
- **Direct Intrahepatic Portocaval Shunt (DIPS)**
- **Transjugular Transcaval Intrahepatic Portosystemic Shunt (TTIPS)**

If the procedure was explicitly performed, return `1` along with an explanation. Otherwise, return `0` with an explanation.

Follow a structured reasoning approach to do the task:

### **Reasoning Steps**
1. **Identify Mentions**: Extract all references to **portosystemic shunt interventions** in the report. Look for terms such as **TIPS, DIPS, TTIPS, transjugular intrahepatic shunt, portocaval shunt, portosystemic shunt placement, shunt revision, or shunt evaluation**.
2. **Confirm the Procedure Type**: Ensure that the report explicitly states that one of the following procedures was performed:
   - **Shunt Placement** (creation of a new TIPS, DIPS, or TTIPS).
   - **Shunt Evaluation** (assessment of an existing shunts function, flow, or patency).
   - **Shunt Revision** (angioplasty, stenting, or any modification to an existing shunt).
3. **Resolve Ambiguities**: If the procedure is only **suggested, planned, considered, or recommended** but not explicitly confirmed as performed, classify it as **not performed**.
4. **Exclude Non-Relevant Procedures**: Ensure that unrelated vascular or hepatic interventions (such as portal vein thrombectomy or general liver angiography) **are not mistaken for portosystemic shunt interventions**.
5. **Handle Uncertainty**: If the report lacks a direct mention of any **portosystemic shunt intervention**, assume it **was not performed**.
6. **Generate JSON Output**: Construct a response in strict JSON format, adhering to the following structure:
```

Prompt for Question 14:

```
Your task is to determine whether **angioplasty or stent placement in the arterial system** was explicitly performed. The **arterial system** includes **any artery in the body**, such as:
- **Coronary arteries** (e.g., coronary angioplasty, coronary stenting).
- **Carotid arteries** (e.g., carotid artery stenting, carotid angioplasty).
- **Peripheral arteries** (e.g., femoral, iliac, popliteal artery interventions).
- **Visceral arteries** (e.g., renal, mesenteric artery stenting).
- **Aortic branches** (e.g., subclavian, vertebral artery stenting).

If the procedure was explicitly performed, return `1` along with an explanation. Otherwise, return `0` with an explanation.

Follow a structured reasoning approach to do the task:

### **Reasoning Steps**
1. **Identify Mentions**: Extract all references to **angioplasty or stent placement** within the **arterial system** from the report. Look for terms such as **angioplasty, percutaneous transluminal angioplasty (PTA), arterial stenting, vascular stenting, endovascular stent placement, or balloon dilation of an artery**.
2. **Confirm the Procedure Type**: Ensure that the report explicitly states that one of the following procedures was performed:
   - **Angioplasty** (balloon dilation of an arterial stenosis).
   - **Stent Placement** (implantation of a stent within an artery).
3. **Resolve Ambiguities**: If the procedure is only **suggested, planned, considered, or recommended** but not explicitly confirmed as performed, classify it as **not performed**.
4. **Exclude Non-Relevant Procedures**: Ensure that interventions performed **in the venous system** (such as venous angioplasty or venous stenting) **are not mistaken for arterial interventions**.
```

```
5. **Handle Uncertainty**: If the report lacks a direct mention of any **arterial angioplasty or stent placement**, assume it **was not performed**.
6. **Generate JSON Output**: Construct a response in strict JSON format, adhering to the following structure:
```

Prompt for Question 15:

```
Your task is to determine whether **angioplasty or stent placement in the venous system** was explicitly performed. The **venous system** includes **any vein in the body**, such as:
- **Central veins** (e.g., superior vena cava (SVC), inferior vena cava (IVC), subclavian, brachiocephalic veins).
- **Peripheral veins** (e.g., iliac, femoral, popliteal, upper extremity veins).
- **Portal or hepatic veins** (e.g., hepatic vein stenting, portal vein angioplasty).
- **Pelvic veins** (e.g., gonadal, ovarian, or internal iliac vein interventions).

If the procedure was explicitly performed, return `1` along with an explanation. Otherwise, return `0` with an explanation.

Follow a structured reasoning approach to do the task:

### **Reasoning Steps**
1. **Identify Mentions**: Extract all references to **venous angioplasty or venous stent placement** from the report. Look for terms such as **venous angioplasty, balloon venoplasty, venous stenting, endovenous stent placement, mechanical luminal disruption, or vein dilation with a balloon**.
2. **Confirm the Procedure Type**: Ensure that the report explicitly states that one of the following procedures was performed:
   - **Venous Angioplasty** (balloon inflation to open a vein or disrupt an obstruction).
   - **Venous Stent Placement** (implantation of a stent within a vein).
3. **Resolve Ambiguities**: If the procedure is only **suggested, planned, considered, or recommended** but not explicitly confirmed as performed, classify it as **not performed**.
4. **Exclude Non-Relevant Procedures**: Ensure that interventions performed **in the arterial system** (such as arterial angioplasty or arterial stenting) **are not mistakenly classified as venous interventions**.
5. **Handle Uncertainty**: If the report lacks direct mention of any **venous angioplasty or stent placement**, assume it **was not performed**.
6. **Generate JSON Output**: Construct a response in strict JSON format, adhering to the following structure:
```

Prompt for Question 16:

```
Your task is to determine whether **stent placement in the carotid artery** was explicitly performed. The carotid arteries include:
- **Common carotid artery (CCA)**
- **Internal carotid artery (ICA)**
- **External carotid artery (ECA)**

If the procedure was explicitly performed, return `1` along with an explanation. Otherwise, return `0` with an explanation.

Follow a structured reasoning approach to do the task:

### **Reasoning Steps**
1. **Identify Mentions**: Extract all references to **stent placement in the carotid artery** from the report. Look for terms such as **carotid stenting, carotid artery stent placement, endovascular stenting of the carotid, carotid angioplasty with stenting, or CAS (Carotid Artery Stenting)**.
2. **Confirm the Procedure Type**: Ensure that the report explicitly states that **a stent was placed in the carotid artery**.
3. **Resolve Ambiguities**: If the procedure is only **suggested, planned, considered, or recommended** but not explicitly confirmed as performed, classify it as **not performed**.
4. **Exclude Non-Relevant Procedures**: Ensure that other **carotid interventions (such as carotid angiography, balloon angioplasty without stent placement, or diagnostic imaging)** are **not mistaken for carotid stenting**.
5. **Handle Uncertainty**: If the report lacks a direct mention of **carotid artery stent placement**, assume it **was not performed**.
```

```
6. **Generate JSON Output**: Construct a response in strict JSON format, adhering to the following structure:
```

Prompt for Question 17:

```
Your task is to determine whether **thrombolytic therapy or thrombectomy** was explicitly performed. These interventions include:

#### **Thrombolytic Therapy** (Clot-Dissolving Medication)
- **Systemic or catheter-directed administration** of thrombolytic agents.
- **Common drugs:**
  - **tPA (tissue plasminogen activator)**
  - **Tenecteplase**
  - **Alteplase**

#### **Thrombectomy** (Clot Removal Procedures)
- **Mechanical thrombectomy**  Direct clot removal using specialized devices (e.g., aspiration catheters, stent retrievers).
- **Balloon thrombectomy**  Clot extraction using a balloon catheter.
- **Pharmacomechanical thrombectomy**  Combination of thrombolytic drugs with mechanical clot disruption.

If the procedure was explicitly performed, return ‘1‘ along with an explanation. Otherwise, return ‘0‘ with an explanation.

Follow a structured reasoning approach to do the task:

### **Reasoning Steps**
1. **Identify Mentions**: Extract all references to **thrombolytic therapy or thrombectomy** from the report. Look for terms such as **tPA administration, thrombolysis, lytic therapy, alteplase, tenecteplase, mechanical thrombectomy, aspiration thrombectomy, balloon thrombectomy, or pharmacomechanical thrombectomy **.
2. **Confirm the Procedure Type**: Ensure that the report explicitly states that **one of the following was performed**:
   - **Thrombolytic therapy** (administration of clot-dissolving drugs).
   - **Thrombectomy** (mechanical or pharmacomechanical clot removal).
3. **Resolve Ambiguities**: If the procedure is only **suggested, planned, considered, or recommended** but not explicitly confirmed as performed, classify it as **not performed**.
4. **Exclude Non-Relevant Procedures**: Ensure that other **vascular interventions (such as angioplasty or stenting)** are **not mistaken for thrombolytic therapy or thrombectomy**.
5. **Handle Uncertainty**: If the report lacks a direct mention of **thrombolytic therapy or thrombectomy**, assume it **was not performed**.
6. **Generate JSON Output**: Construct a response in strict JSON format, adhering to the following structure:
```

Prompt for Question 18:

```
Your task is to determine whether **aortic endograft placement or revision** was explicitly performed. This includes procedures related to:

- **Endovascular Aneurysm Repair (EVAR)**  Placement of an endograft to treat an abdominal aortic aneurysm (AAA).
- **Thoracic Endovascular Aortic Repair (TEVAR)**  Endograft placement in the thoracic aorta.
- **Fenestrated Endovascular Aortic Repair (FEVAR)**  Endograft placement with fenestrations for branch vessels.
- **Endograft Revision**  Any modification, extension, or repair of a previously placed aortic endograft.

If the procedure was explicitly performed, return ‘1‘ along with an explanation. Otherwise, return ‘0‘ with an explanation.

Follow a structured reasoning approach to do the task:

### **Reasoning Steps**
```

```
1. **Identify Mentions**: Extract all references to **aortic endograft placement or revision** from the report
↪ . Look for terms such as **EVAR, TEVAR, FEVAR, aortic stent graft placement, aortic endograft deployment,
↪ endovascular aortic repair, endograft revision, or endograft extension**.
2. **Confirm the Procedure Type**: Ensure that the report explicitly states that **one of the following was
↪ performed**:
   - **Aortic endograft placement** (initial deployment of an aortic stent graft).
   - **Aortic endograft revision** (modification or extension of a prior graft).
3. **Resolve Ambiguities**: If the procedure is only **suggested, planned, considered, or recommended** but
↪ not explicitly confirmed as performed, classify it as **not performed**.
4. **Exclude Non-Relevant Procedures**: Ensure that other **vascular interventions (such as aortic angiography
↪ , open surgical repair, or stenting of non-aortic vessels)** are **not mistaken for aortic endograft
↪ placement or revision**.
5. **Handle Uncertainty**: If the report lacks a direct mention of **aortic endograft placement or revision**,
↪  assume it **was not performed**.
6. **Generate JSON Output**: Construct a response in strict JSON format, adhering to the following structure:
```

Prompt for Question 19:

```
Your task is to determine whether **an emergency embolization** was explicitly performed. Emergency
↪ embolization is defined as the **intravascular administration of an occlusive material to stop or control
↪ active bleeding or hemorrhage**.

#### **Common Indications for Emergency Embolization**
- **Trauma-related bleeding** (e.g., liver, spleen, kidney, or pelvic trauma).
- **Gastrointestinal (GI) bleeding** (e.g., gastric, duodenal, or colonic bleeding).
- **Hemoptysis** (severe lung bleeding).
- **Iatrogenic bleeding** (bleeding caused by medical procedures).
- **Tumor-related hemorrhage** (e.g., bleeding from hypervascular tumors).

#### **Common Embolization Materials**
- **Gelfoam** (temporary occlusion).
- **Onyx** (liquid embolic agent).
- **Beads or microparticles** (used for tumor or GI bleeding control).
- **Coils and plugs** (mechanical occlusion devices).

If the procedure was explicitly performed, return `1` along with an explanation. Otherwise, return `0` with an
↪  explanation.

Follow a structured reasoning approach to do the task:

### **Reasoning Steps**
1. **Identify Mentions**: Extract all references to **emergency embolization** in the report. Look for terms
↪ such as **embolization, endovascular embolization, vascular occlusion, coil embolization, Onyx injection,
↪ Gelfoam, particle embolization, or hemorrhage control procedure**.
2. **Confirm the Procedure Type**: Ensure that the report explicitly states that an **embolization procedure**
↪  was performed **for emergency bleeding control** rather than elective or prophylactic purposes.
3. **Determine the Indication**: Verify that the embolization was performed for **active bleeding, trauma, GI
↪ hemorrhage, hemoptysis, iatrogenic causes, or tumor-related bleeding** rather than for **tumor
↪ devascularization, arteriovenous malformation (AVM) treatment, or preoperative embolization**.
4. **Resolve Ambiguities**: If the report mentions **embolization was considered, planned, or recommended**
↪ but does not confirm that it was **performed**, classify it as **not performed**.
5. **Exclude Non-Relevant Procedures**: Ensure that **angiography, stent placement, thrombectomy, or other
↪ vascular interventions** are **not mistaken for emergency embolization**.
6. **Handle Uncertainty**: If the report lacks a direct mention of **emergency embolization**, assume it **was
↪  not performed**.
7. **Generate JSON Output**: Construct a response in strict JSON format, adhering to the following structure:
```

Prompt for Question 20:

```
Your task is to determine whether **an elective embolization** was explicitly performed. Elective embolization
↪  is defined as the **intravascular administration of an occlusive material to stop or reduce blood flow for
```

```
non-emergency therapeutic purposes** or for the **administration of medication**.

#### **Types of Elective Embolization Procedures**
- **Uterine Artery Embolization (UAE)**  Used to treat uterine fibroids.
- **Uterine Fibroid Embolization (UFE)**  Specific type of UAE for fibroid treatment.
- **Arteriovenous Malformation (AVM) Embolization**  Used to block abnormal blood vessel connections.
- **Varicocele Embolization**  Treats enlarged veins in the scrotum.
- **Gonadal Vein Embolization**  Treats venous insufficiency in reproductive organs.
- **Protective Embolization**  Performed before procedures to prevent complications.

#### **Exclusion Criteria**
- **Transarterial Chemoembolization (TACE) and Transarterial Radioembolization (TARE)** should **not** be considered **elective embolization** **unless protective embolization** was performed.
- **Emergency embolization for active bleeding or trauma** is **not elective** and should be excluded.

If the procedure was explicitly performed, return `1` along with an explanation. Otherwise, return `0` with an explanation.

Follow a structured reasoning approach to do the task:

### **Reasoning Steps**
1. **Identify Mentions**: Extract all references to **elective embolization** from the report. Look for terms such as **uterine artery embolization, fibroid embolization, AVM embolization, varicocele embolization, gonadal vein embolization, protective embolization, or planned vascular occlusion**.
2. **Confirm the Procedure Type**: Ensure that the embolization was performed **for elective (non-emergency) indications**, such as **fibroids, AVMs, or varicoceles**, rather than for **acute hemorrhage control**.
3. **Differentiate from Non-Elective Procedures**:
   - **Exclude emergency embolization** for trauma, GI bleeding, hemoptysis, or tumor-related hemorrhage.
   - **Exclude TACE or TARE unless protective embolization was explicitly mentioned.**
4. **Resolve Ambiguities**: If the report mentions **embolization was considered, planned, or recommended** but does not confirm that it was **performed**, classify it as **not performed**.
5. **Exclude Non-Relevant Procedures**: Ensure that **angiography, venous sclerotherapy, or other vascular interventions** are **not mistaken for elective embolization**.
6. **Handle Uncertainty**: If the report lacks a direct mention of **elective embolization**, assume it **was not performed**.
7. **Generate JSON Output**: Construct a response in strict JSON format, adhering to the following structure:
```

Prompt for Question 21:

```
Your task is to determine whether **a transarterial chemoembolization (TACE) procedure** was explicitly performed. **TACE is defined as the intravascular administration of a chemotherapy agent to treat a tumor.**

#### **Key Criteria for TACE**
- **Must involve intra-arterial chemotherapy delivery** (e.g., doxorubicin, cisplatin, mitomycin C).
- **Must involve arterial embolization** to reduce tumor blood supply.
- **Indications typically include hepatocellular carcinoma (HCC) or metastatic liver tumors.**

#### **Exclusion Criteria**
- **Transarterial Radioembolization (TARE)**  Uses radioactive microspheres instead of chemotherapy.
- **Elective Embolization (e.g., uterine fibroid embolization, AVM embolization)**  Not the same as TACE.
- **Emergency Embolization**  Performed for bleeding, not tumor treatment.

If the procedure was explicitly performed, return `1` along with an explanation. Otherwise, return `0` with an explanation.

Follow a structured reasoning approach to do the task:
### **Reasoning Steps**
1. **Identify Mentions**: Extract all references to **TACE, transarterial chemoembolization, intra-arterial chemotherapy, or embolization for tumor treatment** in the report.
2. **Confirm the Procedure Type**: Ensure the report explicitly states that **chemotherapy was administered
```

```
intra-arterially** and that **embolization was performed as part of the procedure**.
3. **Differentiate from Other Procedures**:
   - **Exclude TARE** (radioembolization with Y-90 microspheres).
   - **Exclude elective embolization for fibroids, AVMs, or varicoceles.**
   - **Exclude emergency embolization for trauma or hemorrhage.**
4. **Resolve Ambiguities**: If the report mentions **TACE was considered, planned, or recommended** but does not confirm that it was **performed**, classify it as **not performed**.
5. **Exclude Non-Relevant Procedures**: Ensure that **biopsy, angiography, or systemic chemotherapy** are **not mistaken for TACE**.
6. **Handle Uncertainty**: If the report lacks a direct mention of **TACE**, assume it **was not performed**.
7. **Generate JSON Output**: Construct a response in strict JSON format, adhering to the following structure:
```

Prompt for Question 22:

```

Your task is to determine whether **a transarterial radioembolization (TARE) procedure** was explicitly performed. **TARE is defined as the intra-arterial administration of radioactive microspheres, most commonly Yttrium-90 (Y90), to treat tumors.**

#### **Key Criteria for TARE**
- **Must involve intra-arterial administration of radioactive material** (e.g., Yttrium-90 microspheres).
- **May include angiographic mapping with or without macroaggregated albumin (MAA) administration** to assess vascular anatomy before Y90 treatment.
- **Typically performed for hepatic tumors (e.g., hepatocellular carcinoma, metastatic liver disease).**

#### **Exclusion Criteria**
- **Transarterial Chemoembolization (TACE)**  Uses **chemotherapy agents** instead of radioactive microspheres .
- **Elective Embolization (e.g., uterine fibroid embolization, AVM embolization)**  Not the same as TARE.
- **Emergency Embolization**  Performed for hemorrhage control, not tumor treatment.

If the procedure was explicitly performed, return ‘1‘ along with an explanation. Otherwise, return ‘0‘ with an  explanation.

Follow a structured reasoning approach to do the task:

### **Reasoning Steps**
1. **Identify Mentions**: Extract all references to **TARE, transarterial radioembolization, Yttrium-90 (Y90) therapy, angiographic mapping, or MAA administration** in the report.
2. **Confirm the Procedure Type**: Ensure the report explicitly states that **radioactive microspheres were administered intra-arterially** or that **angiographic mapping for TARE was performed.**
3. **Differentiate from Other Procedures**:
   - **Exclude TACE** (chemoembolization with doxorubicin, cisplatin, mitomycin C).
   - **Exclude elective embolization for AVMs, fibroids, or varicoceles.**
   - **Exclude emergency embolization for trauma or bleeding.**
4. **Resolve Ambiguities**: If the report mentions **TARE was considered, planned, or recommended** but does not confirm that it was **performed**, classify it as **not performed**.
5. **Exclude Non-Relevant Procedures**: Ensure that **angiography alone, hepatic artery catheterization, or systemic radiation therapy** are **not mistaken for TARE**.
6. **Handle Uncertainty**: If the report is vague or does not explicitly mention of **TARE**, assume it **was not performed**.
7. **Generate JSON Output**: Construct a response in strict JSON format, adhering to the following structure:
```

Prompt for Question 23:

```

Your task is to determine whether an **intravascular procedure was performed** that **does not fit into** any of the following predefined categories:

- **Venous access procedures** (e.g., port placement, exchange, or removal, central venous catheter procedures , dialysis catheter procedures).
- **Dialysis access interventions** (e.g., fistulagrams, graft evaluations, declot procedures for arteriovenous access).
```

```
- **IVC filter placement or removal**.
- **Ablation procedures** (e.g., venous ablation).
- **Stent or stent-graft placement** (arterial or venous).
- **TIPS, DIPS, or other portosystemic shunt procedures**.
- **Embolization procedures** (elective or emergency).
- **Transarterial Chemoembolization (TACE)**.
- **Transarterial Radioembolization (TARE)**.

If a procedure **does not fall under any of these categories** and involves **intravascular access**, it qualifies for a **label of `1`**. Examples of qualifying procedures include:
- **Transjugular liver or renal biopsy**
- **Other intravascular biopsies**
- **Fiducial marker placement using an intravascular approach**

If the procedure was explicitly performed, return `1` along with an explanation. Otherwise, return `0` with an explanation.

Follow a structured reasoning approach to do the task:

### **Reasoning Steps**
1. **Identify Any Intravascular Procedure**:
   - Look for descriptions of procedures that involve catheter-based access to blood vessels.

2. **Check for Exclusion Criteria**:
   - If the procedure is **already classified** under venous access, dialysis interventions, IVC filters, ablations, stents, TIPS, embolization, TACE, or TARE, **do not count it**.

3. **Check for Inclusion Criteria**:
   - If the procedure is **not one of the excluded categories** but still involves **vascular access**, determine if it falls under examples such as **intravascular biopsy (e.g., transjugular liver biopsy, renal biopsy) or fiducial marker placement**.

4. **Resolve Ambiguities**:
   - If the report states that **a procedure was planned or considered** but does not confirm that it was **performed**, classify it as **not performed**.

5. **Ensure No Misclassification**:
   - Verify that **angiography, diagnostic imaging alone, or systemic procedures** are **not mistakenly included** as intravascular interventions.

6. **Generate JSON Output**:
   - Your response **must** be a **valid JSON object** with the following structure:
```

Prompt for Question 24:

```
Your task is to determine whether a **percutaneous biopsy** was performed.

- **Percutaneous biopsy** refers to a biopsy procedure where a needle is inserted **through the skin** to obtain a tissue sample from an organ or mass.
- **Percutaneous biopsy does NOT include intravascular biopsy procedures** such as:
  - **Transjugular liver biopsy**
  - **Transjugular renal biopsy**

If the procedure was explicitly performed, return `1` along with an explanation. Otherwise, return `0` with an explanation.

Follow a structured reasoning approach to do the task:

### **Reasoning Steps**

1. **Identify Any Biopsy Procedure**:
```

```
   - Look for key terms indicating a **biopsy** was performed. Common descriptors include:
     - 'Fine-needle aspiration (FNA)'
     - 'Core needle biopsy (CNB)'
     - 'Image-guided biopsy' (e.g., ultrasound-guided, CT-guided)

2. **Confirm Percutaneous Approach**:
   - Ensure the biopsy was performed **through the skin** and not via a **vascular approach**.
   - If terms like **'transjugular'**, **'intravascular'**, or **'endovascular'** appear, **do not count it** as percutaneous.

3. **Resolve Ambiguities**:
   - If the report states that a **biopsy was considered or planned** but does not confirm it was **performed**, classify it as **not performed**.

4. **Ensure No Misclassification**:
   - If the procedure involves **resection, excision, or surgical biopsy**, it is **not** a percutaneous biopsy and should be excluded.
   - If only **biopsy results** are mentioned but **no procedural details** are given, assume the biopsy is **not documented** in this report.

5. **Generate JSON Output**:
   - Your response **must** be a **valid JSON object** with the following structure:
```

Prompt for Question 25:

```
Your task is to determine whether an **abscess drainage procedure** was performed.

- **Abscess drainage procedures** include:
  - Placement of a drain for an **abscess** or **fluid collection**
  - **Drain revision, repositioning, upsizing, or removal**

- **Exclusions** (Do **not** count the following as abscess drainage):
  - **Biliary drains and tubes**
  - **Cholecystostomy drains and tubes**
  - **Nephrostomy drains and tubes**
  - **Nephroureterostomy drains and tubes**
  - **Chest drains and tubes**

If the procedure was explicitly performed, return `1` along with an explanation. Otherwise, return `0` with an explanation.

Follow a structured reasoning approach to do the task:

### **Reasoning Steps**

1. **Identify Any Drainage Procedure**:
   - Look for terms indicating **drain placement, removal, revision, or repositioning** related to an **abscess or fluid collection**.
   - Common descriptors include:
     - 'Percutaneous abscess drainage'
     - 'Fluid collection drainage'
     - 'Drain placed for abscess'
     - 'Drain repositioning/upsize'

2. **Confirm That It Is an Abscess Drainage Procedure**:
   - Ensure the **drainage target** is an **abscess or fluid collection**, not an excluded category.
   - If the report specifies a **biliary, cholecystostomy, nephrostomy, nephroureterostomy, or chest drain**, **do not count it** as abscess drainage.

3. **Resolve Ambiguities**:
   - If the report states that **drainage was planned or considered** but does **not confirm it was performed
```

```
↪ **, classify it as **not performed**.
   - If **only fluid aspiration is performed without drain placement**, it should **not** be classified as a
↪ drainage procedure.

4. **Ensure No Misclassification**:
   - **Exclude surgical drainage procedures** if they were performed in an **operating room** instead of
↪ radiologically guided drainage.
   - If the **report only mentions drain presence** without confirming **drain placement, revision, or removal
↪ **, assume it is **not documented** in this report.

5. **Generate JSON Output**:
   - Your response **must** be a **valid JSON object** with the following structure:
```

Prompt for Question 26:

```
Your task is to determine whether a **paracentesis or thoracentesis** procedure was performed.

- **Definitions**:
  - **Paracentesis**: A procedure involving **needle drainage of peritoneal fluid (ascites)** from the **
↪ abdomen**.
  - **Thoracentesis**: A procedure involving **needle drainage of pleural fluid (effusion)** from the **chest
↪ (pleural cavity)**.

If the procedure was explicitly performed, return ‘1‘ along with an explanation. Otherwise, return ‘0‘ with an
↪  explanation.

Follow a structured reasoning approach to do the task:

### **Reasoning Steps**

1. **Identify Any Drainage Procedure**:
   - Look for terms indicating **fluid drainage** from the **abdomen (paracentesis)** or **pleural space (
↪ thoracentesis)**.
   - Common descriptors include:
     - 'Paracentesis performed'
     - 'Thoracentesis completed'
     - 'Fluid removed from the peritoneal cavity'
     - 'Pleural effusion drained under ultrasound guidance'

2. **Confirm That It Matches the Definition**:
   - Ensure the report **explicitly states** that **paracentesis or thoracentesis was performed**.
   - If the procedure is **planned or considered but not confirmed as performed**, classify it as **not
↪ performed**.

3. **Exclude Unrelated Drainage Procedures**:
   - **Do not classify as paracentesis/thoracentesis** if:
     - **Fluid aspiration was performed without drainage**.
     - The report describes **abscess drainage, biliary drainage, or nephrostomy**, which are **not**
↪ paracentesis/thoracentesis.
     - A **chest tube** was placed instead of thoracentesis.

4. **Resolve Ambiguities**:
   - If the report **only mentions the presence of fluid (ascites or pleural effusion)** but does not confirm
↪ a **drainage procedure**, classify as **not documented**.
   - If the **procedure attempt failed and no fluid was drained**, classify as **not performed**.

5. **Generate JSON Output**:
   - Your response **must** be a **valid JSON object** with the following structure:
```

Prompt for Question 27:

```
Your task is to determine whether a **chest tube placement** procedure was performed.

- **Definition**:
  - **Chest tube placement** refers to the insertion of a tube into the **pleural space** to drain **air (
↪ pneumothorax), fluid (pleural effusion), blood (hemothorax), pus (empyema), or chyle (chylothorax)**.
  - This is distinct from **thoracentesis**, which involves a needle aspiration without an indwelling tube.

If the procedure was explicitly performed, return ‘1‘ along with an explanation. Otherwise, return ‘0‘ with an
↪  explanation.

Follow a structured reasoning approach to do the task:

### **Reasoning Steps**

1. **Identify Any Tube Placement Procedure**:
   - Look for terms indicating a **chest tube was inserted**.
   - Common descriptors include:
     - 'Chest tube placed'
     - 'Thoracostomy tube inserted'
     - 'Pleural drain inserted'
     - 'Intercostal drain placement'

2. **Confirm That It Matches the Definition**:
   - Ensure that the **tube was actually placed into the pleural space**.
   - **Exclude** procedures that only describe **thoracentesis (needle drainage without a tube)**.

3. **Resolve Ambiguities**:
   - If the report states that **a chest tube was planned or considered** but does **not confirm it was
↪ performed**, classify it as **not performed**.
   - If the **report only mentions the presence of a chest tube** but does not confirm **placement in this
↪ report**, assume it is **not documented** in this instance.

4. **Ensure No Misclassification**:
   - **Do not classify as chest tube placement if:**
     - The procedure was an **aspiration-only thoracentesis** without a tube.
     - The report describes **pleural catheter placement**, which is distinct from a chest tube.
     - The tube was placed **outside the pleural cavity**, such as an abdominal or mediastinal drain.

5. **Generate JSON Output**:
   - Your response **must** be a **valid JSON object** with the following structure:
```

Prompt for Question 28:

```
Your task is to determine whether a **pleurodesis procedure** was performed.

- **Definition**:
  - **Pleurodesis** is a medical procedure that **induces adhesion between the pleural layers** to prevent the
↪  recurrence of pleural effusion or pneumothorax.
  - This is typically achieved by **introducing a chemical agent (e.g., talc, doxycycline, or bleomycin) or
↪ mechanical abrasion into the pleural space**.

If the procedure was explicitly performed, return ‘1‘ along with an explanation. Otherwise, return ‘0‘ with an
↪  explanation.

Follow a structured reasoning approach to do the task:
### **Reasoning Steps**

1. **Identify Any Mention of Pleurodesis**:
   - Look for terms indicating **pleurodesis was performed**.
   - Common descriptors include:
     - 'Chemical pleurodesis with talc/doxycycline performed'
```

```
    - 'Mechanical pleurodesis via abrasion'
    - 'Instillation of pleurodesis agent into pleural space'

2. **Confirm That It Matches the Definition**:
   - Ensure that the **procedure was actually carried out**, not just considered or planned.
   - Verify that **the agent or mechanical technique used for pleurodesis is explicitly stated**.

3. **Resolve Ambiguities**:
   - If the report states that **pleurodesis was planned or considered** but does **not confirm it was
↪ performed**, classify it as **not performed**.
   - If the **report only mentions a prior pleurodesis** without confirming that a **new pleurodesis was
↪ performed**, assume it is **not documented in this instance**.

4. **Ensure No Misclassification**:
   - **Do not classify as pleurodesis if:**
     - The procedure **only describes chest tube placement** without mention of pleurodesis.
     - The **pleural space was drained** (e.g., thoracentesis) but **no pleurodesis agent or abrasion was
↪ applied**.
     - The procedure was a **pleural biopsy or another intervention unrelated to pleurodesis**.

5. **Generate JSON Output**:
   - Your response **must** be a **valid JSON object** with the following structure:
```

Prompt for Question 29:

```
Your task is to determine whether a **procedure involving a biliary drain or biliary stent** was performed.

- **Definition**:
  - **Biliary drain or stent procedures** include:
    - **Percutaneous transhepatic cholangiography (PTC)**
    - **Biliary drain placement, evaluation, or revision**
    - **Biliary stent placement or revision**
  - **Exclusion**:
    - **Cholecystostomy procedures (placement, exchange, or removal) do NOT count as biliary drain or stent
↪ procedures.**

If the procedure was explicitly performed, return `1` along with an explanation. Otherwise, return `0` with an
↪  explanation.

Follow a structured reasoning approach to do the task:
### **Reasoning Steps**

1. **Identify Mentions of a Biliary Drain or Stent Procedure**:
   - Look for phrases such as:
     - 'Percutaneous transhepatic cholangiography (PTC) performed'
     - 'Biliary drain placement/revision/exchange'
     - 'Biliary stent placement/revision'
     - 'Intervention in the biliary system with drain or stent'

2. **Confirm That It Matches the Definition**:
   - Ensure that the procedure was **actually performed**, not just recommended or considered.
   - Verify that **it involves the biliary system** and is **not related to the gallbladder (cholecystostomy)
↪ **.

3. **Resolve Ambiguities**:
   - If the report **mentions a prior biliary drain or stent** but does **not confirm a new procedure was
↪ performed**, classify as **not documented**.
   - If the report **only discusses imaging of the biliary system (e.g., MRCP) without intervention**,
↪ classify as **not performed**.

4. **Exclude Non-Biliary Procedures**:
```

```
   - **Do not classify as a biliary drain or stent procedure if:**
     - The procedure was a **cholecystostomy placement, exchange, or removal**.
     - The report describes **only a diagnostic cholangiogram with no intervention**.
     - The intervention was **related to another organ system (e.g., pancreatic duct stenting, nephrostomy placement, or GI stenting)**.

5. **Generate JSON Output**:
   - Your response **must** be a **valid JSON object** with the following structure:
```

Prompt for Question 30:

```
Your task is to determine whether a **genitourinary drain procedure** was performed.

- **Definition**:
  - **Genitourinary drain procedures** include:
    - **Nephrostomy tube placement, exchange, revision, or removal**
    - **Nephroureteral stent placement, exchange, revision, or removal**

If the procedure was explicitly performed, return `1` along with an explanation. Otherwise, return `0` with an explanation.

Follow a structured reasoning approach to do the task:

### **Reasoning Steps**

1. **Identify Mentions of a Genitourinary Drain Procedure**:
   - Look for phrases such as:
     - 'Nephrostomy tube placed/exchanged/revised/removed'
     - 'Nephroureteral stent placement/exchange/revision/removal'
     - 'Percutaneous nephrostomy procedure performed'
     - 'Intervention involving urinary drainage system'

2. **Confirm That It Matches the Definition**:
   - Ensure that the procedure was **actually performed**, not just recommended or considered.
   - Verify that the procedure was **specific to the genitourinary system**, particularly **kidneys and ureters**.

3. **Resolve Ambiguities**:
   - If the report **mentions a prior nephrostomy tube or stent** but does **not confirm a new procedure**, classify as **not documented**.
   - If the report **only discusses imaging of the genitourinary system (e.g., CT Urogram, MR Urogram) without intervention**, classify as **not performed**.

4. **Exclude Non-Genitourinary Procedures**:
   - **Do not classify as a genitourinary drain procedure if:**
     - The procedure was **related to the bladder or urethra (e.g., Foley catheter placement, suprapubic catheter placement)**.
     - The report describes **only diagnostic imaging without any interventional component**.
     - The intervention was **related to another organ system (e.g., biliary drains, chest tubes, or gastrointestinal stents)**.

5. **Generate JSON Output**:
   - Your response **must** be a **valid JSON object** with the following structure:
```

Prompt for Question 31:

```
Your task is to determine whether an **enterostomy procedure** was performed.

- **Definition**:
  - **Enterostomy procedures** include:
    - **Gastrostomy placement, exchange, revision, or removal**
```

```
    - **Gastrojejunostomy placement, exchange, revision, or removal**
    - **Jejunostomy placement, exchange, revision, or removal**
    - **Ileostomy placement, exchange, revision, or removal**
    - **Cecostomy placement, exchange, revision, or removal**

If the procedure was explicitly performed, return ‘1‘ along with an explanation. Otherwise, return ‘0‘ with an
↪  explanation.

Follow a structured reasoning approach to do the task:

### **Reasoning Steps**

1. **Identify Mentions of an Enterostomy Procedure**:
   - Look for phrases such as:
     - 'Gastrostomy tube placed/exchanged/revised/removed'
     - 'Gastrojejunostomy tube placement/exchange/revision/removal'
     - 'Jejunostomy procedure performed'
     - 'Percutaneous enterostomy tube placement'
     - 'Ileostomy or cecostomy intervention performed'

2. **Confirm That It Matches the Definition**:
   - Ensure that the procedure was **actually performed**, not just recommended or considered.
   - Verify that the procedure was **specific to the gastrointestinal system**, particularly the stomach,
↪ small intestine, or colon.

3. **Resolve Ambiguities**:
   - If the report **mentions a prior enterostomy procedure** but does **not confirm a new procedure**,
↪ classify as **not documented**.
   - If the report **only discusses enteric imaging (e.g., fluoroscopy to check tube placement) without
↪ intervention**, classify as **not performed**.

4. **Exclude Non-Enterostomy Procedures**:
   - **Do not classify as an enterostomy procedure if:**
     - The procedure was **related to the esophagus or rectum (e.g., esophageal stenting, rectal tube
↪ placement)**.
     - The report describes **only diagnostic imaging without any interventional component**.
     - The intervention was **related to another organ system (e.g., biliary or genitourinary procedures)**.

5. **Generate JSON Output**:
   - Your response **must** be a **valid JSON object** with the following structure:
```

Prompt for Question 32:

```
Your task is to determine whether a **cholecystostomy procedure** was performed.

- **Definition**:
  - **Cholecystostomy procedures** include:
    - **Cholecystostomy drain or tube placement**
    - **Cholecystostomy drain or tube exchange**
    - **Cholecystostomy drain or tube revision**
    - **Cholecystostomy drain or tube removal**
    - **Cholecystography (contrast imaging of the gallbladder through a cholecystostomy tube)**

If the procedure was explicitly performed, return ‘1‘ along with an explanation. Otherwise, return ‘0‘ with an
↪  explanation.

Follow a structured reasoning approach to do the task:


### **Reasoning Steps**
```

```
1. **Identify Mentions of a Cholecystostomy Procedure**:
   - Look for phrases such as:
     - 'Cholecystostomy tube placed/exchanged/revised/removed'
     - 'Percutaneous cholecystostomy performed'
     - 'Cholecystography performed via cholecystostomy tube'

2. **Confirm That It Matches the Definition**:
   - Ensure that the procedure was **actually performed**, not just recommended or considered.
   - Verify that the procedure was **specific to the gallbladder**, involving intervention with a drain, tube,
↪  or contrast study.

3. **Resolve Ambiguities**:
   - If the report **mentions a prior cholecystostomy procedure** but does **not confirm a new procedure**,
↪ classify as **not documented**.
   - If the report **only describes gallbladder imaging (e.g., ultrasound, CT, or MRI) without intervention**,
↪  classify as **not performed**.

4. **Exclude Non-Cholecystostomy Procedures**:
   - **Do not classify as a cholecystostomy procedure if:**
     - The procedure was **related to the biliary system but did not involve a cholecystostomy drain or tube (
↪ e.g., biliary stenting, ERCP, PTC)**.
     - The report describes **only gallbladder assessment without intervention**.
     - The intervention was **related to another organ system (e.g., enterostomy or nephrostomy procedures)**.

5. **Generate JSON Output**:
   - Your response **must** be a **valid JSON object** with the following structure:
```

Prompt for Question 33:

```
Your task is to determine whether a **cyst or lymphocele procedure** was performed.

- **Definition**:
  - **Cyst or lymphocele procedures** include:
    - **Aspiration of a cyst or lymphocele**
    - **Drain placement for a cyst or lymphocele**
    - **Drain exchange, revision, or removal for a cyst or lymphocele**
    - **Sclerosis of a cyst or lymphocele (injecting a sclerosing agent to obliterate the lesion)**

If the procedure was explicitly performed, return `1` along with an explanation. Otherwise, return `0` with an
↪  explanation.

Follow a structured reasoning approach to do the task:

### **Reasoning Steps**

1. **Identify Mentions of a Cyst or Lymphocele Procedure**:
   - Look for phrases such as:
     - 'Aspiration of cyst/lymphocele performed'
     - 'Drain placed/exchanged/revised/removed for cyst/lymphocele'
     - 'Sclerotherapy performed for cyst/lymphocele'

2. **Confirm That It Matches the Definition**:
   - Ensure that the procedure was **actually performed**, not just recommended or considered.
   - Verify that the intervention was **specific to a cyst or lymphocele**.

3. **Resolve Ambiguities**:
   - If the report **mentions a prior cyst or lymphocele procedure** but does **not confirm a new procedure**,
↪  classify as **not documented**.
   - If the report **only describes imaging (e.g., ultrasound, CT, or MRI) of a cyst or lymphocele without
↪ intervention**, classify as **not performed**.
```

```
4. **Exclude Non-Cyst/Lymphocele Procedures**:
   - **Do not classify as a cyst or lymphocele procedure if:**
     - The procedure was **related to an abscess or other fluid collection (e.g., abscess drainage, seroma drainage, or pleural effusion drainage)**.
     - The report describes **only diagnostic assessment without an interventional procedure**.
     - The intervention was **related to another organ system (e.g., biliary drainage, peritoneal fluid drainage, nephrostomy, or cholecystostomy procedures)**.

5. **Generate JSON Output**:
   - Your response **must** be a **valid JSON object** with the following structure:
```

Prompt for Question 34:

```
Your task is to determine whether a **nonvascular stent placement** was performed.

- **Definition**:
  - **Nonvascular stents** refer to stents placed in non-blood vessel structures, including:
    - **Esophageal stent placement**
    - **Tracheobronchial (airway) stent placement**
    - **Duodenal stent placement**
    - **Colonic stent placement**

If the procedure was explicitly performed, return ‘1‘ along with an explanation. Otherwise, return ‘0‘ with an explanation.

Follow a structured reasoning approach to do the task:

### **Reasoning Steps**

1. **Identify Mentions of a Nonvascular Stent Placement**:
   - Look for terms such as:
     - 'Esophageal stent placed'
     - 'Tracheal/bronchial stent placement performed'
     - 'Duodenal stent deployed'
     - 'Colonic stent placement completed'

2. **Confirm That It Matches the Definition**:
   - Ensure that the procedure was **actually performed**, not just recommended or planned.
   - Verify that the **stent was placed in a nonvascular structure**.

3. **Resolve Ambiguities**:
   - If the report **mentions a prior nonvascular stent placement** but does **not confirm a new procedure**, classify as **not documented**.
   - If the report **only describes imaging (e.g., X-ray, fluoroscopy, or CT) of a stent without confirming a new placement**, classify as **not performed**.

4. **Exclude Non-Stent and Vascular Stent Procedures**:
   - **Do not classify as nonvascular stent placement if:**
     - The procedure involves **vascular (arterial or venous) stents**, such as in coronary, carotid, iliac, or femoral arteries.
     - The procedure involves **other non-stent interventions**, such as dilation without stent placement.
     - The report describes **only a stent exchange, removal, or revision** without confirming a new stent placement.

5. **Generate JSON Output**:
   - Your response **must** be a **valid JSON object** with the following structure:
```

Prompt for Question 35:

```
Your task is to determine whether a **miscellaneous nonvascular transplant intervention** was performed.
```

```
- **Definition**:
  - A **miscellaneous nonvascular transplant intervention** refers to **procedures performed exclusively on a
↪ transplant organ that do not involve arteries or veins**.
  - The report **must explicitly confirm that the organ is a transplant organ** (not a native organ).
  - **Examples include**:
    - **Stent placement** involving a transplant organ
    - **Balloon plasty procedures**, such as **ureteroplasty**

If the procedure was explicitly performed, return `1` along with an explanation. Otherwise, return `0` with an
↪  explanation.

Follow a structured reasoning approach to do the task:
### **Reasoning Steps**

1. **Identify Mentions of a Transplant Organ**:
   - Look for explicit mentions that the **organ is a transplant**:
     - Phrases like '**transplant kidney**,' '**transplant liver**,' or '**transplant ureter**' confirm that
↪ the organ is not native.
   - If the report does **not** specify that the organ is a **transplant**, classify as **not documented (0)
↪ **.

2. **Identify a Relevant Nonvascular Intervention**:
   - Look for **nonvascular procedures** performed **on the transplant organ**:
     - Stent placements in a transplant organ
     - Balloon plasty procedures (e.g., **ureteroplasty**)
   - **Do NOT include vascular procedures**, such as:
     - **Transplant renal artery stenosis (TRAS) interventions**
     - **Transjugular biopsy procedures**
   - **Do NOT include** general drainage procedures such as:
     - Nephrostomy placement/exchange/removal
     - Nephroureterostomy placement/exchange/removal
     - Biliary tube placement/exchange/removal

3. **Confirm That the Procedure Was Performed**:
   - The procedure must be **explicitly stated as completed**, not just planned or considered.
   - If the report only describes **imaging or evaluation** without confirming an **intervention**, classify
↪ as **not performed**.

4. **Resolve Ambiguities**:
   - If the report **mentions a prior nonvascular transplant intervention** but does **not confirm a new
↪ procedure**, classify as **not documented**.
   - If the procedure is **only vascular in nature**, classify as **not performed**.

5. **Generate JSON Output**:
   - Your response **must** be a **valid JSON object** with the following structure:
```

Prompt for Question 36:

```
Your task is to determine whether a **tumor ablation** procedure was performed.

- **Definition**:
  - A **tumor ablation** is a procedure that destroys tumor tissue using thermal, chemical, or energy-based
↪ methods.
  - **Examples of tumor ablation methods include**:
    - **Radiofrequency ablation (RFA)**
    - **Laser ablation**
    - **Microwave ablation**
    - **Cryoablation**
    - **Ethanol injection/administration**

If the procedure was explicitly performed, return `1` along with an explanation. Otherwise, return `0` with an
```

```
↪ explanation.

Follow a structured reasoning approach to do the task:
### **Reasoning Steps**

1. **Identify Mentions of a Tumor Ablation Procedure**:
   - Look for terms indicating that a **tumor ablation** was **performed**, such as:
     - **'Radiofrequency ablation (RFA) performed'**
     - **'Microwave ablation completed'**
     - **'Cryoablation applied to tumor'**
     - **'Ethanol injection administered'**

2. **Differentiate from Other Procedures**:
   - **Do NOT include** interventions that are **not ablations**, such as:
     - **Surgical tumor resection**
     - **Tumor embolization (TACE or TARE)**
     - **Biopsy procedures**
   - If the report only mentions **imaging, planning, or evaluation** for an ablation **without confirming its
↪ completion**, classify as **not performed**.

3. **Confirm That the Procedure Was Performed**:
   - The procedure must be **explicitly stated as completed**, not just planned or considered.
   - If the report only describes an **intended** or **future** ablation, classify as **not performed**.

4. **Resolve Ambiguities**:
   - If the report mentions a **prior tumor ablation** but does not confirm a new procedure, classify as **not
↪ documented**.
   - If the ablation is only referenced as a **potential treatment option** but was not performed, classify as
↪ **not performed**.

5. **Generate JSON Output**:
   - Your response **must** be a **valid JSON object** with the following structure:
```

Prompt for Question 37:

```
Your task is to determine whether a **pain management procedure** was performed.

- **Definition**:
  - A **pain management procedure** is an interventional procedure aimed at alleviating pain.
  - **Examples of pain management procedures include**:
    - **Steroid injection**
    - **Celiac plexus neurolysis**
    - **Nerve cryoablation**
    - **Periosteal cryoablation**

If the procedure was explicitly performed, return '1' along with an explanation. Otherwise, return '0' with an
↪ explanation.

Follow a structured reasoning approach to do the task:

### **Reasoning Steps**

1. **Identify Mentions of a Pain Management Procedure**:
   - Look for terms indicating that a **pain management intervention** was **performed**, such as:
     - **'Steroid injection administered'**
     - **'Celiac plexus neurolysis performed'**
     - **'Nerve cryoablation completed'**
     - **'Periosteal cryoablation applied'**

2. **Differentiate from Other Procedures**:
   - **Do NOT include** interventions that are **not specifically pain management**, such as:
```

```
    - **General anesthesia or sedation**
    - **Surgical nerve decompression**
    - **Tumor ablation procedures (unless explicitly performed for pain relief)**
  - If the report only mentions **imaging, planning, or evaluation** for a pain management procedure **
↪ without confirming its completion**, classify as **not performed**.

3. **Confirm That the Procedure Was Performed**:
  - The procedure must be **explicitly stated as completed**, not just planned or considered.
  - If the report only describes an **intended** or **future** pain management procedure, classify as **not
↪ performed**.

4. **Resolve Ambiguities**:
  - If the report mentions a **prior pain management procedure** but does not confirm a new one, classify as
↪ **not documented**.
  - If the procedure is only referenced as a **potential treatment option** but was not performed, classify
↪ as **not performed**.

5. **Generate JSON Output**:
  - Your response **must** be a **valid JSON object** with the following structure:
```

Prompt for Question 38:

```
Your task is to determine whether a **Fallopian tube recanalization** was performed.

- **Definition**:
  - **Fallopian tube recanalization (FTR)** is an interventional radiology procedure aimed at reopening
↪ blocked Fallopian tubes to restore fertility.
  - **This procedure involves**:
    - The use of **fluoroscopy and contrast dye** to visualize the Fallopian tubes.
    - **Catheterization** or **balloon inflation** to clear obstructions.

If the procedure was explicitly performed, return '1' along with an explanation. Otherwise, return '0' with an
↪  explanation.

Follow a structured reasoning approach to do the task:

### **Reasoning Steps**

1. **Identify Mentions of a Fallopian Tube Recanalization Procedure**:
  - Look for terms indicating that an **FTR procedure** was performed, such as:
    - **'Fallopian tube recanalization completed'**
    - **'Selective catheterization of the Fallopian tube performed'**
    - **'Balloon dilation of Fallopian tube performed'**

2. **Differentiate from Related Procedures**:
  - **Do NOT include** procedures that are **not Fallopian tube recanalization**, such as:
    - **Hysterosalpingography (HSG) alone** (without intervention)
    - **Salpingectomy (Fallopian tube removal)**
    - **In vitro fertilization (IVF) procedures**
    - **General fertility evaluations or imaging**

3. **Confirm That the Procedure Was Performed**:
  - The procedure must be **explicitly stated as completed**, not just planned or considered.
  - If the report only describes an **intended** or **future** Fallopian tube recanalization, classify as **
↪ not performed**.

4. **Resolve Ambiguities**:
  - If the report mentions a **prior Fallopian tube recanalization** but does not confirm a new one, classify
↪  as **not documented**.
  - If the procedure is only referenced as a **potential treatment option** but was not performed, classify
↪ as **not performed**.
```

```
5. **Generate JSON Output**:
   - Your response **must** be a **valid JSON object** with the following structure:
```

Prompt for Question 39:

```
Your task is to determine whether a **nonvascular, invasive procedure** was performed, while ensuring it **
↪ does not** fall into any of the **excluded categories** listed below.

- **Definition**:
  - A **nonvascular, invasive procedure** involves the insertion of instruments or devices into the body,
↪ either through the skin or a natural orifice, **but does not** involve blood vessels (arteries or veins).

- **Excluded Categories**:
  - **Biopsy (percutaneous or otherwise)**
  - **Abscess drainage & tube checks**
  - **Paracentesis or thoracentesis**
  - **Chest tube placement or pleurodesis**
  - **Biliary interventions** (PTC, biliary drainage/stent procedures)
  - **Tube checks (for any previously placed drainage tubes)**
  - **Genitourinary interventions** (nephrostomy, nephroureteral tube placements/exchanges/removals)
  - **Gastrointestinal interventions** (gastrostomy, gastrojejunostomy, cholecystostomy)
  - **Cyst or lymphocele interventions**
  - **Nonvascular stents** (esophageal, tracheobronchial, duodenal, colonic)
  - **Transplant-related nonvascular interventions**
  - **Tumor ablation (RFA, microwave, cryoablation, ethanol)**
  - **Pain management procedures (nerve blocks, neurolysis, cryoablation)**
  - **Fallopian tube recanalization**
  - **Any intravascular procedure** (angioplasty, stenting, embolization, ablation, dialysis access, venous
↪ access, TIPS, DIPS, IVC filter procedures, TACE, TARE)

If the procedure was explicitly performed, return ‘1‘ along with an explanation. Otherwise, return ‘0‘ with an
↪  explanation.

Follow a structured reasoning approach to do the task:
### **Reasoning Steps**

1. **Identify the Presence of an Invasive Procedure**:
   - Look for descriptions of **instrumentation insertion** into the body through the skin or natural orifices
↪ .
   - Common indicators include:
     - **Percutaneous intervention**
     - **Endoscopic intervention**
     - **Device placement**
     - **Surgical instrument insertion**

2. **Check If the Procedure Falls Into an Excluded Category**:
   - Compare the procedure against the **list of excluded interventions**.
   - If the procedure **matches any exclusion**, classify as **not qualifying (return ‘0‘)**.

3. **Determine If the Procedure Is a Nonvascular, Invasive Procedure Not Listed as Excluded**:
   - If it **does not** involve **arteries or veins** and is **not in the exclusion list**, classify as **
↪ qualifying (return ‘1‘)**.
   - Examples of qualifying procedures:
     - **Foreign body removal from a nonvascular structure**
     - **Fiducial marker placement (nonvascular target, such as lung or prostate)**
     - **Percutaneous decompression of a non-abscess fluid collection (not cyst/lymphocele-related)**

4. **Resolve Ambiguities**:
   - If the report **only mentions a history of a procedure but no new procedure was performed**, classify as
↪ **not documented (‘0‘)**.
```

```
   - If the procedure is **described as planned but not yet performed**, classify as **not performed ('0')**.

5. **Generate JSON Output**:
   - Your response **must** be a **valid JSON object** with the following structure:
```

Appendix F.3. Example Report and Labels

Example Report

```
Pre-procedure diagnosis: Cholelithiasis
Post-procedure diagnosis: Same
Indication: Cholelithiasis
Additional clinical history: None

Complications: No immediate complications.

IMPRESSION:

*       Extensive cholelithiasis with partial removal of gallstones.
*       Stricture at the cystic duct, status post balloon dilation.
*       Appropriately positioned internal/external biliary drain.

Plan:

*       Maintain biliary drain to gravity drainage. Flush twice daily to ensure tube patency.
*       Plan for staged gallstone lithotripsy and extraction.
PROCEDURE SUMMARY:
- Transhepatic cholangiogram via the existing access
- Cholangioscopy with gallstone lithotripsy and extraction.
- Additional procedure(s): Internal/external biliary drain placement.

PROCEDURE DETAILS:

Pre-procedure
Consent: Informed consent for the procedure including risks, benefits and alternatives was obtained and time-
↪ out was performed prior to the procedure.
Preparation: The site was prepared and draped using maximal sterile barrier technique including cutaneous
↪ antisepsis.

Anesthesia/sedation
Level of anesthesia/sedation: General anesthesia
Anesthesia/sedation administered by: Anesthesiology
Total intra-service sedation time (minutes): See anesthesia flow sheet.

Cholangiogram, cholangioscopy, and gallstone lithotripsy/extraction.
Local anesthesia was administered. Contrast was injected through the indwelling biliary drain. A guidewire was
↪  passed through the gallbladder, cystic and common bile ducts, into the small bowel. Sequential dilation
↪ was performed, until ultimately a peel-away sheath was inserted into the gallbladder. A cholangioscope was
↪ advanced over the wire into the gallbladder. Electrohydraulic lithotripsy and basket retrieval of
↪ gallstones were performed. Due to extensive gallstone burden of large size and limited electrohydraulic
↪ lithotripsy devices, the visualized gallstones were incompletely extracted. A cholangiogram was
↪ subsequently performed through the peel-away sheath, demonstrating a focal area of stenosis at the cystic
↪ duct. Balloon dilation of the cystic and common duct was performed, with resolution of the previous area of
↪  stenosis.. A new internal/external biliary drainage catheter was placed, and the location of the distal
↪ tip was confirmed to be within small bowel following contrast injection..
Initial cholangiogram findings: Extensive gallstones burden. Contrast flows across the cystic duct into the
↪ duodenum.
New biliary drain: Cook internal/external biliary drain
New biliary drain diameter (French): 14
New biliary drain modifications: None
Final cholangiogram findings: Cholelithiasis. Internal/external biliary drain in place with contrast draining
↪ into the duodenum.
```

```
External catheter securement:  Non-absorbable suture

Contrast
Contrast agent: Omnipaque 300
Contrast volume (mL): 20

Radiation Dose
Fluoroscopy time (minutes): 17.0
Reference air kerma (mGy): 185.1
Kerma area product (uGy-m2): 2685.2

Additional Details
Additional description of procedure: None
Equipment details: None
Specimens removed: None
Estimated blood loss (mL): Less than 10
```

Response from Claude-3.5-Haiku with CoT Prompting

Response for Question 1:

```
"No explicit mention or evidence of CTA or intra-operative cone-beam CT with contrast in the radiology report.
↪  Procedures were fluoroscopy-guided with contrast cholangiography."
```

Response for Question 2:

```
"No mention or evidence of MR Angiography (MRA) in the radiology report. The procedure involved endoscopic
↪ gallstone lithotripsy, cholangiography, and biliary drain placement using fluoroscopy."
```

Response for Question 3:

```
"No noninvasive vascular lab study (duplex ultrasound, color flow study, or PVR) was mentioned or performed in
↪  this radiology report, which focused on an endoscopic gallstone procedure."
```

Response for Question 4:

```
"No cardiac imaging study was performed in this radiology report, which details an endoscopic gallstone
↪ lithotripsy and biliary intervention procedure."
```

Response for Question 5:

```
"No arteriography was performed. The procedure was an endoscopic gallstone lithotripsy with biliary
↪ cholangiography, using contrast in the biliary system, not an arterial study."
```

Response for Question 6:

```
"No venography was performed. The procedure was an endoscopic gallstone lithotripsy with cholangiography using
↪  biliary contrast, not a venous imaging study."
```

Response for Question 7:

```
"No dialysis access evaluation was performed in this radiology report, which describes a biliary procedure
↪ involving gallstone lithotripsy and drain placement."
```

Response for Question 8:

```
"No carotid artery imaging was performed or mentioned in this radiology report, which describes an endoscopic
↪ gallstone lithotripsy procedure with biliary interventions."
```

Response for Question 9:

```
"No central venous catheter procedure was performed. The report details a biliary drain procedure with
↪ cholangioscopy and gallstone lithotripsy."
```

Response for Question 10:

```
"No IVC filter placement or removal was mentioned in the radiology report. The procedure focused on gallstone
↪ lithotripsy and biliary drain placement."
```

Response for Question 11:

```
"The radiology report describes a biliary intervention for gallstone management, with no mention of any venous
↪  ablation procedure"
```

Response for Question 12:

```
"No dialysis access intervention was performed. This report describes a biliary procedure involving gallstone
↪ lithotripsy and biliary drain placement."
```

Response for Question 13:

```
"No portosystemic shunt intervention was performed. The procedure was a biliary intervention involving
↪ gallstone lithotripsy and biliary drain placement."
```

Response for Question 14:

```
"The report describes an endoscopic gallstone lithotripsy with biliary drain placement and balloon dilation of
↪  biliary ducts, but no arterial angioplasty or stent placement was performed."
```

Response for Question 15:

```
"The radiology report describes a biliary intervention with no venous angioplasty or stent placement performed
↪ "
```

Response for Question 16:

```
"The radiology report describes an endoscopic gallstone lithotripsy and biliary drain placement procedure,
↪ with no mention of carotid artery stenting."
```

Response for Question 17:

```
"No thrombolytic therapy or thrombectomy was performed in this biliary intervention procedure focused on
↪ gallstone management"
```

Response for Question 18:

```
"No evidence of aortic endograft placement or revision in this radiology report. The procedure was a biliary
↪ intervention involving gallstone lithotripsy and drain placement."
```

Response for Question 19:

```
"No emergency embolization was performed. The procedure was a gallstone lithotripsy with biliary drain
↪ placement."
```

Response for Question 20:

```
"No evidence of elective embolization in this radiology report. The procedure was a gallstone lithotripsy and
↪ biliary intervention."
```

Response for Question 21:

```
"The report describes an endoscopic gallstone lithotripsy and biliary drain procedure. There is no mention of
↪ TACE, chemotherapy administration, or tumor embolization. The procedure focuses on gallstone removal and
↪ biliary drainage, which is entirely unrelated to transarterial chemoembolization."
```

Response for Question 22:

```
"This report describes an endoscopic gallstone lithotripsy with biliary drain placement. No evidence of
↪ transarterial radioembolization (TARE) was present."
```

Response for Question 23:

```
"This is a biliary procedure involving cholangioscopy and gallstone lithotripsy, with no intravascular
↪ intervention performed"
```

Response for Question 24:

```
"No percutaneous biopsy was performed in this radiology report. The procedure was focused on gallstone
↪ lithotripsy, cholangioscopy, and biliary drain placement."
```

Response for Question 25:

```
"This is a biliary drain placement procedure, which is explicitly excluded from abscess drainage procedures
↪ according to the defined criteria."
```

Response for Question 26:

```
"No paracentesis or thoracentesis was performed. The report describes an endoscopic gallstone lithotripsy with
↪  biliary drain placement, which is a different type of procedure."
```

Response for Question 27:

```
"This radiology report describes a biliary drain placement procedure, not a chest tube placement. The tube was
↪  placed in the biliary system, not the pleural space."
```

Response for Question 28:

```
"No pleurodesis procedure was performed. The report describes an endoscopic gallstone lithotripsy and biliary
↪ drain placement, with no mention of any pleural space intervention."
```

Response for Question 29:

```
"An internal/external biliary drain was explicitly placed during the procedure, with detailed documentation of
↪  drain type, size, and placement confirmation"
```

Response for Question 30:

```
"The report describes a biliary intervention with biliary drain placement, which is not a genitourinary drain
↪ procedure. No nephrostomy tubes or nephroureteral stents were placed."
```

Response for Question 31:

```
"The report describes a biliary intervention with cholangioscopy and drain placement, but no enterostomy
↪ procedure was performed."
```

Response for Question 32:

```
"While a biliary drain was placed, this was not a specific cholecystostomy procedure. The intervention
↪ involved cholangioscopy, gallstone lithotripsy, and biliary drain placement, but did not meet the strict
↪ definition of a cholecystostomy."
```

Response for Question 33:

```
"This report describes a biliary intervention for gallstones, with no cyst or lymphocele procedure performed.
↪ The procedure involved cholangioscopy, gallstone lithotripsy, and biliary drain placement."
```

Response for Question 34:

```
"An internal/external biliary drain (nonvascular stent) was placed during the procedure, specifically a Cook
↪ 14 French biliary drain"
```

Response for Question 35:

```
"No transplant organ was mentioned; this was a procedure on a native biliary system"
```

Response for Question 36: **False Positive**

```
"No tumor ablation procedure was performed. The report details an endoscopic gallstone lithotripsy and biliary
↪  drain placement procedure."
```

Response for Question 37:

```
"No pain management procedure was performed. The report details a gallstone lithotripsy and biliary
↪ intervention without any specific pain management intervention."
```

Response for Question 38:

```
"The radiology report describes a biliary procedure involving gallstone lithotripsy and does not mention any
↪ Fallopian tube intervention."
```

Response for Question 39: **False Negative**

```
"The procedure involves biliary interventions, specifically biliary drainage and drain placement, which are
↪ explicitly excluded from qualifying as a nonvascular, invasive procedure."
```
